# Moderate exercise and chronic stress produce counteractive effects on different areas of the brain by acting through various neurotransmitter receptor subtypes: A hypothesis

**DOI:** 10.1186/1742-4682-3-33

**Published:** 2006-09-23

**Authors:** Suptendra N Sarbadhikari, Asit K Saha

**Affiliations:** 1 TIFAC-CORE in Biomedical Technology, Amrita Vishwa Vidyapeetham, Amritapuri 690525, India; 2 School of Electrical and Information Engineering, University of South Australia, Mawson Lakes Campus, South Australia 5095, Australia

## Abstract

**Background:**

Regular, "moderate", physical exercise is an established non-pharmacological form of treatment for depressive disorders. Brain lateralization has a significant role in the progress of depression. External stimuli such as various stressors or exercise influence the higher functions of the brain (cognition and affect). These effects often do not follow a linear course. Therefore, nonlinear dynamics seem best suited for modeling many of the phenomena, and putative global pathways in the brain, attributable to such external influences.

**Hypothesis:**

The general hypothesis presented here considers only the nonlinear aspects of the effects produced by "moderate" exercise and "chronic" stressors, but does not preclude the possibility of linear responses. In reality, both linear and nonlinear mechanisms may be involved in the final outcomes. The well-known neurotransmitters serotonin (5-HT), dopamine (D) and norepinephrine (NE) all have various receptor subtypes. The article hypothesizes that 'Stress' increases the activity/concentration of some particular subtypes of receptors (designated nt_s_) for each of the known (and unknown) neurotransmitters in the right anterior (RA) and left posterior (LP) regions (cortical and subcortical) of the brain, and has the converse effects on a different set of receptor subtypes (designated nt_h_). In contrast, 'Exercise' increases nt_h _activity/concentration and/or reduces nt_s _activity/concentration in the LA and RP areas of the brain. These effects may be initiated by the activation of Brain Derived Neurotrophic Factor (BDNF) (among others) in exercise and its suppression in stress.

**Conclusion:**

On the basis of this hypothesis, a better understanding of brain neurodynamics might be achieved by considering the oscillations caused by single neurotransmitters acting on their different receptor subtypes, and the temporal pattern of recruitment of these subtypes. Further, appropriately designed and planned experiments will not only corroborate such theoretical models, but also shed more light on the underlying brain dynamics.

## Background

Regular, "moderate", physical exercise is a non-pharmacological form of adjunctive treatment for depressive disorders. External stimuli such as various stressors or exercise influence the higher functions of the brain (cognition and affect). These effects often do not follow a linear course. Even though exercise itself can be seen as a stressor, in moderate doses it has been shown to reduce the effects of other stressors. To explain our hypothesis better, we need to elaborate on certain concepts – encompassing a wide range of biological and mathematical domains – of stress, depression, exercise, neurotransmitters along with their receptor subtypes, brain lateralization and nonlinear dynamics. All these concepts (and their interactions) are discussed broadly in the following paragraphs in this section. The hypothesis is based on the numerous published data obtained from experimental research, and on logical assumptions made where experimental data are not yet available. We have tried to thread together the gems (some key studies) of experimental evidence presented in Table 1[1-27]. The approach is more akin to systems biology (generalization) than to detailed characterization of any particular pathway of exercise and stress actions. The reader is encouraged to ponder over the items in Table 1 before going through the rest of this section for elucidation of the relevant concepts. A highly focused "linear" thought process may not be conducive to comprehending the underlying essential nonlinearities in our proposed model.

**Table 1:** Highlights of some relevant literature (abbreviations expanded in the text)

**Areas, Author (Year)**	**Summary**	**Relevance**
**A. Origin of the idea** Sarbadhikari (1995a) [1]	Exercise reduces behavioral and EEG effects of stress	Mechanism to be determined
**B. Stress and lateralization** Mandal et al. (1996), Atchely *et al*. (2003); Neveu and Merlot (2003); Yurgelun-Todd & Ross (2006) [2&6]	Definite lateralization effects observed for affect and stress	Stress acts in a lateralized fashion; lateralization of emotion in depression; lateralized effects of stress may act at cellular levels
**C. Chaos and nonlinear dynamics in depression** Toro *et al*. (1999); Levine *et al*. (2000); Thomasson *et al*. (2000); Jeong (2002) [7–10]	Chaotic oscillations in the brain may account for many conditions including depression, where there is proven correlation between clinical and electrophysiological dimensions, and associations between clinical remission and bifurcation are present	Chaotic oscillations form one of the mechanisms for depression
**D. Exercise, lateralization and nonlinear dynamics** Petruzzello *et al*. (2001); Kyriazis (2003) [11,12]	Exercise influences affective responsiveness by regional brain activation and also increases physiological complexity in the brain	Exercise acts in a lateralized fashion and increases complexity, unlike stress
**E. Nonlinear dynamics linking various physiological and pathological processes** Sarbadhikari and Chakrabarty (2001); Glass (2001); Savi (2005) [13–15]	Nonlinear dynamics can be the underlying commonalty between depression, exercise and lateralization	Depression, exercise and lateralization may all be nonlinearly linked; Stress and Exercise may operate counteractively through the same systems
**F. Neurotransmitter receptor subtypes have varied functions and distributions** Tecott (2000); Pediconi *et al*. (1993); Bortolozzi *et al *(2003); Xu *et al*. (2005); Fukumoto *et al*. (2005), et al [16–22]	Receptor subtypes for all neurotransmitters; asymmetric distribution of acetylcholine and monoamine receptors in mammalian brain	Same neurotransmitter may act in opposing ways by binding with different receptor subtypes; asymmetric distributions of various neurotransmitters are possible in the brain
**G. Cellular level interactions involving BDNF and CREB** Cotman *et al*. (2002); Garoflos *et al*. (2005) [23, 24]	BDNF increases with Exercise and decreases with Stress; phosphorylation of the transcription factor CREB and increased BDNF expression are positively correlated	BDNF and CREB may be intermediaries for activating the various receptor subtypes
**H. Integrating hypothesis** Shenal *et al*. (2003) [25]	LF, RF and RP interactions in the brain are responsible for the manifestation of stress effects	LA/RA/RP/LP quadratic interactions could give rise to cross-coupling of the systems
**I. Detailed expositions** Sarbadhikari (2005a, b) [26, 27]	Depressive and dementive disorders can be caused by nonlinear disturbances in lateralization	Stress and Exercise may operate counteractively through the same systems

Broadly: "Stress" refers to the mental or physical condition resulting from various disturbing physical, emotional, or chemical factors ("stressors"), which can be environmental or anthropogenic, and lead to a behavior or outcome that is commonly labeled "depressive". The effects of the stressors on the body constitute the "stress response", which may be measured by behavioral, biochemical, and genetic modifications. "Anxiety" may be defined as the emotional discomfort associated with "stress". "Depression" denotes a spectrum of disorders affecting many aspects of human physiology, and can be precipitated by various psychological (*e.g*., mental trauma), biophysical (*e.g*., loss of organ or function and genetic predisposition) and social (*e.g*., loss of job) stressors. However, under-diagnosis in general medical practice is quite common [1].

Depression (including its various subtypes) is a common global disorder. Apart from newer pharmacotherapeutic management, some non-pharmacological interventions also play a significant part in its alleviation [1]. Regular, "moderate" physical exercise forms a pillar of such treatment. Our hypothesis concerns general mechanisms that give rise to the effects of exercise along with stress.

Cerebral hemispheric lateralization alludes to the localization of brain function on either the right or left sides of the brain, and is an important factor in the progress of depression [2]. Incidentally, this lateralization is not confined to only the cerebral cortices, but also to the subcortical structures. A recent paper [3] indicates that mood state may be differentiated by lateralization of brain activation in fronto-limbic regions. The interpretation of fMRI (functional magnetic resonance imaging) studies in bipolar disorder is limited by the choice of regions of interest, medication effects, comorbidity, and task performance. These studies suggest that there is a complex alteration in regions important for neural networks underlying cognition and emotional processing in bipolar disorder. However, measuring changes in specific brain regions does not identify how these neural networks are affected. New techniques for analyzing fMRI data are needed in order to resolve some of these issues and identify how changes in neural networks relate to cognitive and emotional processing in bipolar disorder.

The relationship between exercise and stress is not a simple one. As succinctly pointed out by Mastorakos and Pavlatou [4]: "Exercise represents a physical stress that challenges homeostasis. In response to this stressor, the autonomic nervous system and hypothalamus-pituitary-adrenal axis are known to react and participate in the maintenance of homeostasis and the development of physical fitness. This includes elevation of cortisol and catecholamines in plasma. However, physical conditioning is associated with a reduction in pituitary-adrenal activation in response to exercise." In our present model, we shall start at the point at which chronic moderate exercise has already led to the "baseline adaptive changes" and behaves in a different way from any other stressor. In future modifications, changes in the model's threshold for exhibiting this particular (bimodal) behavior can also be incorporated. This bimodal or hormetic response is characterized by low dose stimulation, high dose inhibition, resulting in either a J-shaped or an inverted U-shaped (nonlinear) dose response. A chemical pollutant or toxin or radiation showing hormesis therefore has the opposite effect in small doses to that in large doses. Therefore, we can assume regular moderate exercise as the mild, repeated "stressful" stimulation (which is good for health). While excessive and prolonged stress (as in heavy exercise) can lead to depression, mild and irregular (non-linearly applied, hormetic) stress can actually improve depression. Radak *et al*. [28] extend the hormesis theory to include reactive oxygen species (ROS). They further suggest that the beneficial effects of regular exercise are partly based on the ROS-generating capacity of exercise, which is in the stimulation range of ROS production. Therefore, they suggest that exercise-induced ROS production plays a role in the induction of antioxidants, DNA repair and protein degrading enzymes, resulting in decreases in the incidence of oxidative stress-related diseases.

External stimuli such as various stressors or exercise influence the higher brain functions, i.e., cognition and affect. These effects often do not follow a linear course. In nonlinear dynamics the rate of change of any variable cannot be written as a linear function of the other variables. Therefore, it may be better suited to modeling many phenomena, and putative global pathways, in the brain, that are attributable to such influences [7,8,12-15].

**Figure 1:**
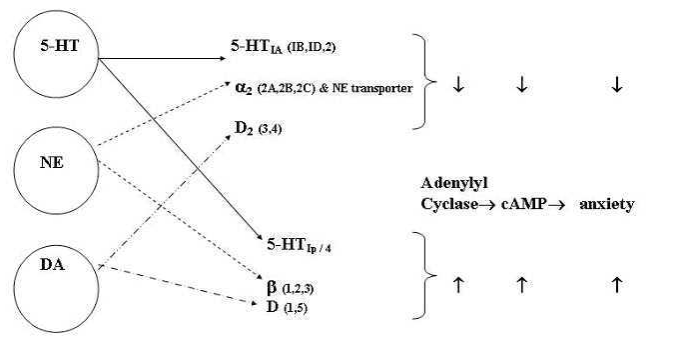
Typical example of complementary action of some neurotransmitter receptor subtypes. Key: DA: Dopamine; NE: Norepinephrine; 5HT: 5-Hydroxytryptamine or Serotonin.

Neurotransmitters convey the information to be passed and processed through some 10^14 ^to 10^16 ^interconnections linking approximately 10^10 ^to 10^11 ^neurons in the human brain. Each of the many neurotransmitters (including as yet unidentified ones) acts through a receptor, which in general will have numerous subtypes [16]. The same neurotransmitter acting through two different receptor subtypes may have opposing actions. Most psychotropic drugs exert their therapeutic effects through various neurotransmitters, mainly through specific receptor subtypes. Some neurotransmitter receptor subtype interactions are depicted in Figure 1. It may be noted that 5-HT_2 _class receptors couple to Gq/G11 and do not primarily signal through cAMP pathways. Similarly, 5-HT_3 _receptors are ligand-coupled ion channels and do not primarily signal through cAMP as Figure 1 might seem to suggest. However, this only proves the existence of additional intracellular pathways such as the Gq/G11 coupled intracellular calcium/protein kinase C pathway, and also highlights the fact that signaling is much more complex than this model allows. Our oversimplification is essential for trying to grasp the overall complexity of all possible (known and as yet unknown) underlying mechanisms of the brain. The basic purpose of this figure is to show that (irrespective of the mechanisms of action) any neurotransmitter is capable of exerting opposing effects (e.g., increasing anxiety or 'anxiogenesis' and decreasing anxiety or 'anxiolysis') by acting through its diverse receptor subtypes.

Interestingly, there is a greater right-sided EEG abnormality in depression owing to impaired cerebral lateralization [2]. Therapeutically, too, better antidepressant results are obtained with non-dominant unilateral electroconvulsive shock. It is generally believed that "affect" processing is a right hemisphere (RH) function. It is also believed that RH dysfunction is characteristic of depressive illness. Both these beliefs are oversimplified because the relationship between affect processing and affective illness, in terms of intra- and inter-hemispheric role-play, is not straightforward. There is exchange of information and action between the two hemispheres (inter-hemispheric, *i.e*., between left and right; intra-hemispheric *i.e*., between anterior and posterior; and also cross-hemispheric coupling *i.e*., similarities between the left anterior and right posterior quadrants). Very broadly, a sad mood is a function of positive coupling (stimulation) between the left posterior and right anterior areas and/or negative coupling (depression) between the left anterior and right posterior areas of the brain [2].

Brain functions are lateralized to the right or the left sides and there are observed differences in the expression of neurotransmitter receptor subtypes [16-22]. Some of these results [21] are supported by a meta-analysis of various studies reported in the literature. Neuroanatomical asymmetries are known to be present in the human brain, and disturbed neurochemical asymmetries have also been reported in the brains of patients with schizophrenia [22]. Not only neuroanatomical but also neurochemical evidence supports the loss or reversal of normal asymmetry of the temporal lobe in schizophrenia, which might be due to a disruption of the neurodevelopmental processes involved in hemispheric lateralization.

Neuropsychological research provides a useful framework for studying emotional problems such as depression and their correlates. Shenal *et al*. [25] review several prominent neuropsychological theories focusing on functional neuroanatomical systems of emotion and depression, including those that describe cerebral asymmetries in emotional processing. Following their review, they present a model comprising three neuroanatomical divisions (left frontal, right frontal and right posterior) and corresponding neuropsychological emotional sequelae within each quadrant. It is proposed that dysfunction in any of these quadrants could lead to symptomatology consistent with a diagnosis of depression. Their model combines theories of arousal, lateralization and functional cerebral space and lends itself to scientific investigation. Shenal *et al*. [25] conclude: 'As the existing literature appears to be somewhat confusing and controversial, an increased precision for the diagnostic term "depression" may afford a better understanding of this emotional construct. Future research projects and innovative neuropsychological models may help to form a better understanding of depression.' Their proposed model 'combines theories of arousal, lateralization, and functional cerebral space to better understand these distinct clinical pictures, and it should be noted that these regions may be differentially activated following various therapies to depressive symptomatology.' However, their excellent neuropsychological model does not take into account the different neurotransmitter receptor subtype distribution and functions.

The theory of dynamical systems ("chaos theory") allows one to describe the change in a system's macroscopic behavior as a bifurcation in the underlying dynamics. From the example of depressive syndrome, a correspondence can be demonstrated between clinical and electro-physiological dimensions and the association between clinical remission and reorganization of brain dynamics (*i.e*., bifurcation). Thomasson *et al*. [9] discuss the relationship between mind and brain in respect of the question of normality versus pathology in psychiatry on the basis of their experimental study.

Neuropharmacological investigations aimed at understanding the electrophysiological correlates between drug effects and action potential trains have usually involved the analysis of firing rate and bursting activity. Di Mascio *et al*. [29] selectively altered the neural circuits that provide inputs to dopaminergic neurons in the ventral tegmental area and investigated the corresponding electrophysiological correlates by nonlinear dynamic analysis. The nonlinear prediction method combined with Gaussian-scaled surrogate data showed that the structure in the time-series corresponding to the electrical activity of these neurons, extracellularly recorded *in vivo*, was chaotic. A decrease in chaos of these dopaminergic neurons was found in a group of rats treated with 5,7-dihydroxytryptamine, a neurotoxin that selectively destroys serotonergic terminals. The chaos content of the ventral tegmental area dopaminergic neurons in the control group, and the decrease of chaos in the lesioned group, cannot be explained in terms of standard characteristics of neuronal activity (firing rate, bursting activity). Moreover, the control group showed a positive correlation between the density-power-spectrum of the interspike intervals (ISIs) and the chaos content measured by nonlinear prediction S score; this relationship was lost in the lesioned group. It was concluded that the impaired serotonergic tone induced by 5,7-dihydroxytryptamine reduces the chaotic behavior of the dopaminergic cell-firing pattern while retaining many standard ISI characteristics. However, some difficulties remain. There is a suspicion that the determinism in the EEG may be too high-dimensional to be detected with current methods. Previously [30], ISIs of dopamine neurons recorded in the substantia nigra were predicted partially on the basis of their immediate prior history. These data support the hypothesis that the sequence-dependent behavior of dopamine neurons arises in part from interactions with forebrain structures. ISI sequences recorded from unlesioned rats demonstrated maximum predictability when an average of 3.7 prior events were incorporated into the forecasting algorithm, implying a physiological process, the "depth" of history-dependence of which is approximately 600–800 ms.

It has been repeatedly confirmed that the brain acts nonlinearly, especially when complex interactions are required, as in cognition or affect processing. In a cognitive study [31], although the nonlinear measures ranged in the middle field compared to the number of significant contrasts, they were the only ones that were partially successful in discriminating among the mental tasks. In another cognitive study [32], initial increase in complexity of both episodic and semantic information was associated with right inferior frontal activation; further increase in complexity was associated with left dorsolateral activation. This implies that frontal activation during retrieval is a non-linear function of the complexity of the retrieved information.

A broader view of stress is that not only do dramatic stressful events exact a toll, but also the many events of daily life elevate the activities of physiological systems and cause some measure of wear and tear. This wear and tear has been termed "allostatic load" [33], and it reflects the impact not only of life experiences but also of genetic load (predisposition); individual habits reflecting items such as diet, exercise and substance abuse, and developmental experiences that set life-long patterns of behavior and physiological reactivity. Hormones and neurotransmitters associated with stress and allostatic load protect the body in the short term and promote adaptation, but in the long run allostatic load causes changes in the body that lead to disease. These have been observed particularly in the immune system and the brain.

Zheng *et al*. [34] state that exercise has beneficial effects on mental health in depressed sufferers; however, the mechanisms underlying these effects remained unresolved. These authors found that (1) exercise reversed the harmful effects of chronic unpredictable stress on mood and spatial performance in rats and (2) the behavioral changes induced by exercise and/or chronic unpredictable stress might be associated with hippocampal brain-derived neurotrophic factor (BDNF) levels. Also, the HPA (hypothalamus-pituitary-adrenal axis) system might play different roles in the two processes. BDNF is the most widely-distributed trophic factor in the brain and participates in neuronal growth, maintenance and use-dependent plasticity mechanisms such as long-term potentiation (LTP) and learning. Huang *et al*. [35] observed that compulsive treadmill exercise with pre-familiarization acutely up-regulates expression of the BDNF gene in rat hippocampus. Duman [36] states that stress and depression decrease neurotrophic factor expression and neurogenesis in the brain, and that antidepressant treatment blocks or reverses these effects. In contrast, exercise and enriched environment increase neurotrophic support and neurogenesis, which could contribute to blockading the effects of stress and aging and produce antidepressant effects. BDNF, in turn, exerts its effects through the formation/suppression of specific neurons, neurotransmitters, and receptor subtypes. Another study [37] corroborates the substantial data implicating common pathways involving neurotransmitter action through neurotrophic factors in the regulation of neural stem cells. This transmitter-mediated neurotrophic pathway could be altered by environmental factors including enriched environment, exercise, stress, and drug abuse. The most notable neurotransmitters in this context are serotonin (5-HT), glutamate and gamma-amino-butyric acid (GABA). There is ample evidence that enhancement of neurotrophic support and associated augmentation of synaptic plasticity and function may form the basis for antidepressant efficacy [38]. Although depression is not a homogeneous disorder, some commonalty may be expected in the final common pathway for all forms of depression. Incidentally, exercise has various other effects (as mentioned in the limitations section), which are not discussed here. Also, exercise, as a stimulus, is dependent on its timing (what time of day it is performed), frequency (how many times a day, or a week) and content (aerobic, weight bearing and so on). The very fact that these parameters can be varied is a stimulus itself, and variations in them have physical influences on brain function, including upregulation of trophic factors such as GDNF (glial cell line-derived neurotrophic factor), FGF-2 (Fibroblast growth factor-2), or BDNF [39].

The beneficial role of exercise is evident in many neurodegenerative disorders [40]. Despite the paucity of human research, basic animal models and clinical data overwhelmingly support the notion that exercise treatment is a major protective factor against neurodegeneration of various etiologies. The final common pathway of degradation is clearly related to oxidative stress, nitrosative stress, glucocorticoid dysregulation, inflammation and amyloid deposition. Exercise training may be a major protective factor but in the absence of clinical guidelines, its prescription and success with treatment adherence remain elusive. In the present model, Moderate Exercise: 3.0 – 6.0 METs (3.5 – 7.0 kcal/min) [41] is assumed for the purpose of modeling.

Freeman [42] believes that the search for simple rules is one good reason for using the tools of chaos theory to model neural functions. The present effort is to integrate these clues theoretically in order to gain a better overview of the interactions of stress and exercise inside the brain. The next section describes our preliminary hypothesis based on some experimental evidence.

To sum up, it is not known whether the complex dynamics are an essential feature or if they are secondary to internal feedback and environmental fluctuations [13]. Because of the complexity of biological systems and the huge jumps in scale from a single ionic channel to the cell to the organ to the organism, all computer models will be gross approximations to the real system for the foreseeable future. There is a rich fMRI literature on affect, stress and depression and this, together with a wealth of preclinical data, will enable the very general model proposed in this paper to be refined in the future. At present, our concern is to determine whether a broadly testable nonlinear dynamic model can be elaborated and to outline the preliminary experiments required to validate it. Only after this task is completed will detailed refinement, producing a more practically helpful model, become appropriate. It may be noted that the basic purpose of the model is to provide direction for experimental research, since there is a paucity of real life data, which we feel to be essential for understanding the precise role of neurotransmitter receptor subtypes in different areas of the brain.

## The Hypothesis

### Introduction

The preliminary general model described here is based on the assumptions that (a) some neurotransmitter cascade (primarily nonlinear) affects the whole brain in a lateralized fashion, and (b) with more prolonged exercise, more favorable receptor subtypes are recruited for all the neurotransmitters involved.

From our previous studies [1,43,44], we found that the deleterious behavioral effects of stress were less pronounced in the "exercised and stressed" animals, and the beneficial effects became more pronounced with time (more prolonged exercise), as indicated by the results of the behavioral tests.

Let us cite another example of (nonlinear) interactions among diverse neurotransmitters. Di Mascio *et al*. [29] showed that a 5-HT antagonist impairs serotoninergic tone, which in turn reduces the chaotic behavior of dopaminergic cell firing patterns in the brain. Another study by Toro *et al*. [7] included pharmacological modification of neurotransmitter pathways, electroconvulsive therapy (ECT), sleep deprivation, psychosurgery, electrical stimulation through cerebral electrodes, and repetitive transcranial magnetic stimulation (rTMS). Stemming from a pathophysiological model that portrays the brain as an open, complex and nonlinear system, a common mechanism of action has been attributed to all therapies. This report suggests that the isomorphism among therapies is related to their ability to help the CNS deactivate cortical-subcortical circuits that are dysfunctionally coupled. These circuits are self-organized among the neurons of their informational (rapid conduction) and modulating (slow conduction) subsystems. The following speculative overview is based on the aforementioned review and the detailed expositions by Sarbadhikari [26,27]. Disease specific genes (and *ipso facto *proteins) give rise to individual variations in different receptor subtype populations (endowment). This is the basis of pharmacogenomic (individualized) therapy in modern medicine. Each of the conditions mentioned here leads to a (primarily nonlinear) imbalance among the endowed receptor subtype populations (in specific areas of the brain) and tilts the final common pathway in favor of depression or elation. In the previous section, we mentioned some reports that support this view.

**Figure 2:**
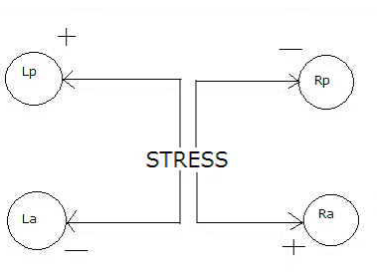
Schematic diagram of stress activity within the brain.

It may be surmised that some neurotransmitter cascade (nonlinear or a combination of linear and nonlinear) takes place in different areas of the whole brain, and, with more prolonged exercise, more favorable receptor subtypes are recruited. Stress leads to more left sided (RH or right hemisphere) psychomotor activity, which causes RH inhibition (negative valence), ultimately giving rise to sadness or more negative interpretation. Very broadly, a sad mood is a function of positive coupling (stimulation) between the left posterior and right anterior areas and/or negative coupling (depression) between the left anterior and right posterior areas of the brain. Figure 2 presents a schematic diagram of stress activity within the brain.

Moderate exercise, in contrast, causes more right-sided (psychomotor) activity leading to LH (left hemisphere) inhibition (positive valence), facilitating assertiveness or less negative interpretation. However, a happy mood is broadly a function of positive coupling (stimulation) between the right posterior and left anterior areas and/or negative coupling (depression) between the right anterior and left posterior areas of the brain [25]. These couplings are at least partly caused by the activation of Brain Derived Neurotrophic Factor (BDNF) in exercise and the suppression of BDNF in stress [22]. BDNF activation and phosphorylation of the cAMP response element binding (CREB) protein are also positively correlated [23]. Further, the results of a study [45] are consistent with the hypothesis that decreased expression of BDNF and possibly other growth factors contributes to depression and that upregulation of BDNF plays a role in the actions of antidepressant treatment. Another study [46] suggests that in the frontal cortex and amygdala of mice, caffeic acid can attenuate the down-regulation of BDNF transcription that results from stressful conditions. Recently, investigators [47] have shown that imipramine (IMI) and metyrapone (MET) significantly elevate the BDNF mRNA level in the hippocampus and cerebral cortex. Joint administration of IMI and MET induces a more potent increase BDNF gene expression in both the examined brain regions compared to the treatment with either drug alone.

**Figure 3:**
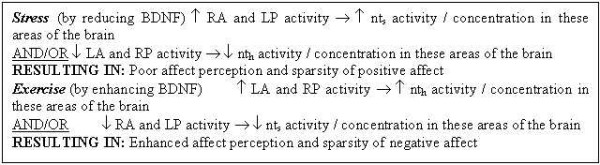
Some putative biochemical aspects of the hypothesis.

This article assumes a particular subtype of neurotransmitter receptor (designated nt_s_), which could be 5-HT_4_, D_1,5_, β adrenoceptors or yet unidentified types. These are mostly responsible for the "anxiogenic" effects, leading to a "sad" mood. These are assumed to be more active/concentrated in the RA (right anterior) and LP (left posterior) quadrants of the brain. Another set of receptor subtypes (designated nt_h_) are assumed for 5-HT_1A_, D_2_, NE or yet unidentified transporters. These are mostly responsible for the "anxiolytic" effects, giving rise to a "happy" mood, and are assumed to be more active/concentrated in the LA (left anterior) and RP (right posterior) quadrants of the brain. The predictions of this proposed model are indicated in Figure 3.

To explain our hypothesis better, we briefly revisit the first two models from our previous work [43].

### Model-1: The effects of stress on the four different quadrants of the brain

The terms *L*_*a*_, *L*_*p*_, *R*_*a *_and *R*_*p *_represent the release of neurotransmitters from the axons of neurons in the four different quadrants of the brain (left anterior, left posterior, right anterior and right posterior) due to stress activity. The left-posterior and right-anterior areas of the brain are positively activated by stress whereas left-anterior and right-posterior quadrants are negatively activated by a feedback mechanism.

*St *denotes the stress activity; α_*i*_(*i *= 1,2,3,4) denotes the activation rates and γ_*i*_(*i *= 1,2,3,4) the natural degradation rates; *n*_*j*_(*j *= 2,3) are the Hill coefficients; and *h *is the threshold value of the neuron. The corresponding model may be defined by:

ddt(Lp)=α1(St)−γ1(Lp)ddt(La)=α2hn2+(St)n2−γ2(La)ddt(Rp)=α3hn3+(St)n3−γ3(Rp)ddt(Ra)=α4(St)−γ4(Ra)ddt(St)=f(St)     {1}
 MathType@MTEF@5@5@+=feaafiart1ev1aaatCvAUfKttLearuWrP9MDH5MBPbIqV92AaeXatLxBI9gBaebbnrfifHhDYfgasaacH8akY=wiFfYdH8Gipec8Eeeu0xXdbba9frFj0=OqFfea0dXdd9vqai=hGuQ8kuc9pgc9s8qqaq=dirpe0xb9q8qiLsFr0=vr0=vr0dc8meaabaqaciaacaGaaeqabaqabeGadaaakeaafaqaaeqbbaaaaeaadaWcaaqaaiabdsgaKbqaaiabdsgaKjabdsha0baacqGGOaakcqWGmbatcqWGWbaCcqGGPaqkcqGH9aqpcqaHXoqydaWgaaWcbaGaeGymaedabeaakiabcIcaOiabdofatjabdsha0jabcMcaPiabgkHiTiabeo7aNnaaBaaaleaacqaIXaqmaeqaaOGaeiikaGIaemitaWKaemiCaaNaeiykaKcabaWaaSaaaeaacqWGKbazaeaacqWGKbazcqWG0baDaaGaeiikaGIaemitaWKaemyyaeMaeiykaKIaeyypa0ZaaSaaaeaacqaHXoqydaWgaaWcbaGaeGOmaidabeaaaOqaaiabdIgaOnaaCaaaleqabaGaemOBa42aaSbaaWqaaiabikdaYaqabaaaaOGaey4kaSIaeiikaGIaem4uamLaemiDaqNaeiykaKYaaWbaaSqabeaacqWGUbGBdaWgaaadbaGaeGOmaidabeaaaaaaaOGaeyOeI0Iaeq4SdC2aaSbaaSqaaiabikdaYaqabaGccqGGOaakcqWGmbatcqWGHbqycqGGPaqkaeaadaWcaaqaaiabdsgaKbqaaiabdsgaKjabdsha0baacqGGOaakcqWGsbGucqWGWbaCcqGGPaqkcqGH9aqpdaWcaaqaaiabeg7aHnaaBaaaleaacqaIZaWmaeqaaaGcbaGaemiAaG2aaWbaaSqabeaacqWGUbGBdaWgaaadbaGaeG4mamdabeaaaaGccqGHRaWkcqGGOaakcqWGtbWucqWG0baDcqGGPaqkdaahaaWcbeqaaiabd6gaUnaaBaaameaacqaIZaWmaeqaaaaaaaGccqGHsislcqaHZoWzdaWgaaWcbaGaeG4mamdabeaakiabcIcaOiabdkfasjabdchaWjabcMcaPaqaamaalaaabaGaemizaqgabaGaemizaqMaemiDaqhaaiabcIcaOiabdkfasjabdggaHjabcMcaPiabg2da9iabeg7aHnaaBaaaleaacqaI0aanaeqaaOGaeiikaGIaem4uamLaemiDaqNaeiykaKIaeyOeI0Iaeq4SdC2aaSbaaSqaaiabisda0aqabaGccqGGOaakcqWGsbGucqWGHbqycqGGPaqkaeaadaWcaaqaaiabdsgaKbqaaiabdsgaKjabdsha0baacqGGOaakcqWGtbWucqWG0baDcqGGPaqkcqGH9aqpcqWGMbGzcqGGOaakcqWGtbWucqWG0baDcqGGPaqkaaGaaCzcaiaaxMaadaGadeqaaiabigdaXaGaay5Eaiaaw2haaaaa@B164@

Irrespective of the source, the effects of stress are cumulative, but we assume that they cannot accumulate indefinitely – there must be a point of 'sustainability'. Here, we consider this stage as a suicidal point(*K*). Therefore, effects of stress can go up to a saturation stage (*K*) beyond which a suicidal tendency will develop. It may be noted that whether a person not doing exercise will actually commit suicide depends on the chaotic or unpredictable behavior of the system in the individual.

To the best of the authors' knowledge, there currently exists no mathematical model to explain stress dynamics clearly. As a first attempt we have considered the Volterra equation to represent stress dynamics. The justification for this selection is that there exists a saturation level in the Volterra equation. As such we can choose f(St)=α5(St){1−(St)K}
 MathType@MTEF@5@5@+=feaafiart1ev1aaatCvAUfKttLearuWrP9MDH5MBPbIqV92AaeXatLxBI9gBaebbnrfifHhDYfgasaacH8akY=wiFfYdH8Gipec8Eeeu0xXdbba9frFj0=OqFfea0dXdd9vqai=hGuQ8kuc9pgc9s8qqaq=dirpe0xb9q8qiLsFr0=vr0=vr0dc8meaabaqaciaacaGaaeqabaqabeGadaaakeaacqWGMbGzcqGGOaakcqWGtbWucqWG0baDcqGGPaqkcqGH9aqpcqaHXoqydaWgaaWcbaGaeGynaudabeaakiabcIcaOiabdofatjabdsha0jabcMcaPmaacmqabaGaeGymaeJaeyOeI0YaaSaaaeaacqGGOaakcqWGtbWucqWG0baDcqGGPaqkaeaacqWGlbWsaaaacaGL7bGaayzFaaaaaa@4408@, where (*K*) is the carrying capacity for stress and α_5 _is the intrinsic growth rate of stress. Hence system {1} becomes

ddt(Lp)=α1(St)−γ1(Lp)ddt(La)=α2hn2+(St)n2−γ2(La)ddt(Rp)=α3hn3+(St)n3−γ3(Rp)ddt(Ra)=α4(St)−γ4(Ra)ddt(St)=α5(St){1−(St)K}     {2}
 MathType@MTEF@5@5@+=feaafiart1ev1aaatCvAUfKttLearuWrP9MDH5MBPbIqV92AaeXatLxBI9gBaebbnrfifHhDYfgasaacH8akY=wiFfYdH8Gipec8Eeeu0xXdbba9frFj0=OqFfea0dXdd9vqai=hGuQ8kuc9pgc9s8qqaq=dirpe0xb9q8qiLsFr0=vr0=vr0dc8meaabaqaciaacaGaaeqabaqabeGadaaakeaafaqaaeqbbaaaaeaadaWcaaqaaiabdsgaKbqaaiabdsgaKjabdsha0baacqGGOaakcqWGmbatdaWgaaWcbaGaemiCaahabeaakiabcMcaPiabg2da9iabeg7aHnaaBaaaleaacqaIXaqmaeqaaOGaeiikaGIaem4uamLaemiDaqNaeiykaKIaeyOeI0Iaeq4SdC2aaSbaaSqaaiabigdaXaqabaGccqGGOaakcqWGmbatdaWgaaWcbaGaemiCaahabeaakiabcMcaPaqaamaalaaabaGaemizaqgabaGaemizaqMaemiDaqhaaiabcIcaOiabdYeamnaaBaaaleaacqWGHbqyaeqaaOGaeiykaKIaeyypa0ZaaSaaaeaacqaHXoqydaWgaaWcbaGaeGOmaidabeaaaOqaaiabdIgaOnaaCaaaleqabaGaemOBa42aaSbaaWqaaiabikdaYaqabaaaaOGaey4kaSIaeiikaGIaem4uamLaemiDaqNaeiykaKYaaWbaaSqabeaacqWGUbGBdaWgaaadbaGaeGOmaidabeaaaaaaaOGaeyOeI0Iaeq4SdC2aaSbaaSqaaiabikdaYaqabaGccqGGOaakcqWGmbatdaWgaaWcbaGaemyyaegabeaakiabcMcaPaqaamaalaaabaGaemizaqgabaGaemizaqMaemiDaqhaaiabcIcaOiabdkfasnaaBaaaleaacqWGWbaCaeqaaOGaeiykaKIaeyypa0ZaaSaaaeaacqaHXoqydaWgaaWcbaGaeG4mamdabeaaaOqaaiabdIgaOnaaCaaaleqabaGaemOBa42aaSbaaWqaaiabiodaZaqabaaaaOGaey4kaSIaeiikaGIaem4uamLaemiDaqNaeiykaKYaaWbaaSqabeaacqWGUbGBdaWgaaadbaGaeG4mamdabeaaaaaaaOGaeyOeI0Iaeq4SdC2aaSbaaSqaaiabiodaZaqabaGccqGGOaakcqWGsbGudaWgaaWcbaGaemiCaahabeaakiabcMcaPaqaamaalaaabaGaemizaqgabaGaemizaqMaemiDaqhaaiabcIcaOiabdkfasnaaBaaaleaacqWGHbqyaeqaaOGaeiykaKIaeyypa0JaeqySde2aaSbaaSqaaiabisda0aqabaGccqGGOaakcqWGtbWucqWG0baDcqGGPaqkcqGHsislcqaHZoWzdaWgaaWcbaGaeGinaqdabeaakiabcIcaOiabdkfasnaaBaaaleaacqWGHbqyaeqaaOGaeiykaKcabaWaaSaaaeaacqWGKbazaeaacqWGKbazcqWG0baDaaGaeiikaGIaem4uamLaemiDaqNaeiykaKIaeyypa0JaeqySde2aaSbaaSqaaiabiwda1aqabaGccqGGOaakcqWGtbWucqWG0baDcqGGPaqkdaGadeqaaiabigdaXiabgkHiTmaalaaabaGaeiikaGIaem4uamLaemiDaqNaeiykaKcabaGaem4saSeaaaGaay5Eaiaaw2haaaaacaWLjaGaaCzcamaacmqabaGaeGOmaidacaGL7bGaayzFaaaaaa@BE1E@

The non-trivial steady state solution of the system {2} is given by

S0={α1Kγ1,α2γ2(hn2+Kn2),α3γ3(hn3+Kn3),α4Kγ4,K}T     {3}
 MathType@MTEF@5@5@+=feaafiart1ev1aaatCvAUfKttLearuWrP9MDH5MBPbIqV92AaeXatLxBI9gBaebbnrfifHhDYfgasaacH8akY=wiFfYdH8Gipec8Eeeu0xXdbba9frFj0=OqFfea0dXdd9vqai=hGuQ8kuc9pgc9s8qqaq=dirpe0xb9q8qiLsFr0=vr0=vr0dc8meaabaqaciaacaGaaeqabaqabeGadaaakeaacqWGtbWudaahaaWcbeqaaiabicdaWaaakiabg2da9maacmqabaWaaSaaaeaacqaHXoqydaWgaaWcbaGaeGymaedabeaakiabdUealbqaaiabeo7aNnaaBaaaleaacqaIXaqmaeqaaaaakiabcYcaSmaalaaabaGaeqySde2aaSbaaSqaaiabikdaYaqabaaakeaacqaHZoWzdaWgaaWcbaGaeGOmaidabeaakiabcIcaOiabdIgaOnaaCaaaleqabaGaemOBa42aaSbaaWqaaiabikdaYaqabaaaaOGaey4kaSIaem4saS0aaWbaaSqabeaacqWGUbGBdaWgaaadbaGaeGOmaidabeaaaaGccqGGPaqkaaGaeiilaWYaaSaaaeaacqaHXoqydaWgaaWcbaGaeG4mamdabeaaaOqaaiabeo7aNnaaBaaaleaacqaIZaWmaeqaaOGaeiikaGIaemiAaG2aaWbaaSqabeaacqWGUbGBdaWgaaadbaGaeG4mamdabeaaaaGccqGHRaWkcqWGlbWsdaahaaWcbeqaaiabd6gaUnaaBaaameaacqaIZaWmaeqaaaaakiabcMcaPaaacqGGSaaldaWcaaqaaiabeg7aHnaaBaaaleaacqaI0aanaeqaaOGaem4saSeabaGaeq4SdC2aaSbaaSqaaiabisda0aqabaaaaOGaeiilaWIaem4saSeacaGL7bGaayzFaaWaaWbaaSqabeaacqWGubavaaGccaWLjaGaaCzcamaacmqabaGaeG4mamdacaGL7bGaayzFaaaaaa@6A8F@

The dimensionless form of {2} can be expressed as {4}:

ddtx1=β1x5−δ1x1ddtx2=β21+x5n2−δ2x2ddtx3=β31+x5n3−δ3x3ddtx4=β4x5−δ4x4ddtx5=β5x5(1−x5ℜ)     {4}
 MathType@MTEF@5@5@+=feaafiart1ev1aaatCvAUfKttLearuWrP9MDH5MBPbIqV92AaeXatLxBI9gBaebbnrfifHhDYfgasaacH8akY=wiFfYdH8Gipec8Eeeu0xXdbba9frFj0=OqFfea0dXdd9vqai=hGuQ8kuc9pgc9s8qqaq=dirpe0xb9q8qiLsFr0=vr0=vr0dc8meaabaqaciaacaGaaeqabaqabeGadaaakeaafaqaaeqbbaaaaeaadaWcaaqaaiabdsgaKbqaaiabdsgaKjabdsha0baacqWG4baEdaWgaaWcbaGaeGymaedabeaakiabg2da9iabek7aInaaBaaaleaacqaIXaqmaeqaaOGaemiEaG3aaSbaaSqaaiabiwda1aqabaGccqGHsislcqaH0oazdaWgaaWcbaGaeGymaedabeaakiabdIha4naaBaaaleaacqaIXaqmaeqaaaGcbaWaaSaaaeaacqWGKbazaeaacqWGKbazcqWG0baDaaGaemiEaG3aaSbaaSqaaiabikdaYaqabaGccqGH9aqpdaWcaaqaaiabek7aInaaBaaaleaacqaIYaGmaeqaaaGcbaGaeGymaeJaey4kaSIaemiEaG3aa0baaSqaaiabiwda1aqaaiabd6gaUnaaBaaameaacqaIYaGmaeqaaaaaaaGccqGHsislcqaH0oazdaWgaaWcbaGaeGOmaidabeaakiabdIha4naaBaaaleaacqaIYaGmaeqaaaGcbaWaaSaaaeaacqWGKbazaeaacqWGKbazcqWG0baDaaGaemiEaG3aaSbaaSqaaiabiodaZaqabaGccqGH9aqpdaWcaaqaaiabek7aInaaBaaaleaacqaIZaWmaeqaaaGcbaGaeGymaeJaey4kaSIaemiEaG3aa0baaSqaaiabiwda1aqaaiabd6gaUnaaBaaameaacqaIZaWmaeqaaaaaaaGccqGHsislcqaH0oazdaWgaaWcbaGaeG4mamdabeaakiabdIha4naaBaaaleaacqaIZaWmaeqaaaGcbaWaaSaaaeaacqWGKbazaeaacqWGKbazcqWG0baDaaGaemiEaG3aaSbaaSqaaiabisda0aqabaGccqGH9aqpcqaHYoGydaWgaaWcbaGaeGinaqdabeaakiabdIha4naaBaaaleaacqaI1aqnaeqaaOGaeyOeI0IaeqiTdq2aaSbaaSqaaiabisda0aqabaGccqWG4baEdaWgaaWcbaGaeGinaqdabeaaaOqaamaalaaabaGaemizaqgabaGaemizaqMaemiDaqhaaiabdIha4naaBaaaleaacqaI1aqnaeqaaOGaeyypa0JaeqOSdi2aaSbaaSqaaiabiwda1aqabaGccqWG4baEdaWgaaWcbaGaeGynaudabeaakmaabmGabaGaeGymaeJaeyOeI0YaaSaaaeaacqWG4baEdaWgaaWcbaGaeGynaudabeaaaOqaaiabgYricdaaaiaawIcacaGLPaaaaaGaaCzcaiaaxMaadaGadeqaaiabisda0aGaay5Eaiaaw2haaaaa@9D4E@

Where

x1=h−1(Lp),x2=h−1(La),x3=h−1(Rp),x4=h−1(Ra),x5=h−1(St),β1=α1h2,β2=α2h−n2+1,β3=α3h−n3+1,β4=α4h2,β5=α5h2,δ1=γ1h2,δ2=γ2h2,δ3=γ3h2,δ4=γ4h2,ℜ=Kh−1,τ=h−2t     {5}
 MathType@MTEF@5@5@+=feaafiart1ev1aaatCvAUfKttLearuWrP9MDH5MBPbIqV92AaeXatLxBI9gBaebbnrfifHhDYfgasaacH8akY=wiFfYdH8Gipec8Eeeu0xXdbba9frFj0=OqFfea0dXdd9vqai=hGuQ8kuc9pgc9s8qqaq=dirpe0xb9q8qiLsFr0=vr0=vr0dc8meaabaqaciaacaGaaeqabaqabeGadaaakeaafaqaaeWabaaabaGaemiEaG3aaSbaaSqaaiabigdaXaqabaGccqGH9aqpcqWGObaAdaahaaWcbeqaaiabgkHiTiabigdaXaaakiabcIcaOiabdYeamnaaBaaaleaacqWGWbaCaeqaaOGaeiykaKIaeiilaWIaemiEaG3aaSbaaSqaaiabikdaYaqabaGccqGH9aqpcqWGObaAdaahaaWcbeqaaiabgkHiTiabigdaXaaakiabcIcaOiabdYeamnaaBaaaleaacqWGHbqyaeqaaOGaeiykaKIaeiilaWIaemiEaG3aaSbaaSqaaiabiodaZaqabaGccqGH9aqpcqWGObaAdaahaaWcbeqaaiabgkHiTiabigdaXaaakiabcIcaOiabdkfasnaaBaaaleaacqWGWbaCaeqaaOGaeiykaKIaeiilaWIaemiEaG3aaSbaaSqaaiabisda0aqabaGccqGH9aqpcqWGObaAdaahaaWcbeqaaiabgkHiTiabigdaXaaakiabcIcaOiabdkfasnaaBaaaleaacqWGHbqyaeqaaOGaeiykaKIaeiilaWIaemiEaG3aaSbaaSqaaiabiwda1aqabaGccqGH9aqpcqWGObaAdaahaaWcbeqaaiabgkHiTiabigdaXaaakiabcIcaOiabdofatjabdsha0jabcMcaPiabcYcaSaqaaiabek7aInaaBaaaleaacqaIXaqmaeqaaOGaeyypa0JaeqySde2aaSbaaSqaaiabigdaXaqabaGccqWGObaAdaahaaWcbeqaaiabikdaYaaakiabcYcaSiabek7aInaaBaaaleaacqaIYaGmaeqaaOGaeyypa0JaeqySde2aaSbaaSqaaiabikdaYaqabaGccqWGObaAdaahaaWcbeqaaiabgkHiTiabd6gaUnaaBaaameaacqaIYaGmaeqaaSGaey4kaSIaeGymaedaaOGaeiilaWIaeqOSdi2aaSbaaSqaaiabiodaZaqabaGccqGH9aqpcqaHXoqydaWgaaWcbaGaeG4mamdabeaakiabdIgaOnaaCaaaleqabaGaeyOeI0IaemOBa42aaSbaaWqaaiabiodaZaqabaWccqGHRaWkcqaIXaqmaaGccqGGSaalcqaHYoGydaWgaaWcbaGaeGinaqdabeaakiabg2da9iabeg7aHnaaBaaaleaacqaI0aanaeqaaOGaemiAaG2aaWbaaSqabeaacqaIYaGmaaGccqGGSaalcqaHYoGydaWgaaWcbaGaeGynaudabeaakiabg2da9iabeg7aHnaaBaaaleaacqaI1aqnaeqaaOGaemiAaG2aaWbaaSqabeaacqaIYaGmaaGccqGGSaalaeaacqaH0oazdaWgaaWcbaGaeGymaedabeaakiabg2da9iabeo7aNnaaBaaaleaacqaIXaqmaeqaaOGaemiAaG2aaWbaaSqabeaacqaIYaGmaaGccqGGSaalcqaH0oazdaWgaaWcbaGaeGOmaidabeaakiabg2da9iabeo7aNnaaBaaaleaacqaIYaGmaeqaaOGaemiAaG2aaWbaaSqabeaacqaIYaGmaaGccqGGSaalcqaH0oazdaWgaaWcbaGaeG4mamdabeaakiabg2da9iabeo7aNnaaBaaaleaacqaIZaWmaeqaaOGaemiAaG2aaWbaaSqabeaacqaIYaGmaaGccqGGSaalcqaH0oazdaWgaaWcbaGaeGinaqdabeaakiabg2da9iabeo7aNnaaBaaaleaacqaI0aanaeqaaOGaemiAaG2aaWbaaSqabeaacqaIYaGmaaGccqGGSaalcqGHCeIWcqGH9aqpcqWGlbWscqWGObaAdaahaaWcbeqaaiabgkHiTiabigdaXaaakiabcYcaSiabes8a0jabg2da9iabdIgaOnaaCaaaleqabaGaeyOeI0IaeGOmaidaaOGaemiDaqhaaiaaxMaacaWLjaWaaiWabeaacqaI1aqnaiaawUhacaGL9baaaaa@E152@

The time dependent general solution of stress in dimensionless form is given by

x5(τ)=x5(τ0)ℜx5(τ0)+{ℜ−x5(τ0)}eβ5τ     {6}
 MathType@MTEF@5@5@+=feaafiart1ev1aaatCvAUfKttLearuWrP9MDH5MBPbIqV92AaeXatLxBI9gBaebbnrfifHhDYfgasaacH8akY=wiFfYdH8Gipec8Eeeu0xXdbba9frFj0=OqFfea0dXdd9vqai=hGuQ8kuc9pgc9s8qqaq=dirpe0xb9q8qiLsFr0=vr0=vr0dc8meaabaqaciaacaGaaeqabaqabeGadaaakeaacqWG4baEdaWgaaWcbaGaeGynaudabeaakiabcIcaOiabes8a0jabcMcaPiabg2da9maalaaabaGaemiEaG3aaSbaaSqaaiabiwda1aqabaGccqGGOaakcqaHepaDdaWgaaWcbaGaeGimaadabeaakiabcMcaPiabgYricdqaaiabdIha4naaBaaaleaacqaI1aqnaeqaaOGaeiikaGIaeqiXdq3aaSbaaSqaaiabicdaWaqabaGccqGGPaqkcqGHRaWkcqGG7bWEcqGHCeIWcqGHsislcqWG4baEdaWgaaWcbaGaeGynaudabeaakiabcIcaOiabes8a0naaBaaaleaacqaIWaamaeqaaOGaeiykaKIaeiyFa0Naemyzau2aaWbaaSqabeaacqaHYoGydaWgaaadbaGaeGynaudabeaaliabes8a0baaaaGccaWLjaGaaCzcamaacmqabaGaeGOnaydacaGL7bGaayzFaaaaaa@5C1B@

Where *x*_5_(τ_0_) > 0 is the initial stress when τ = τ_0_.

The time dependent solutions of *L*_*p *_and *R*_*a *_in dimensionless form are given by

x1=e−δ1τ∫{β1x5(τ0)ℜeδ1τx5(τ0)+[ℜ−x5(τ0)e−β5τ}dτ+CLpe−δ1τ     {7}
 MathType@MTEF@5@5@+=feaafiart1ev1aaatCvAUfKttLearuWrP9MDH5MBPbIqV92AaeXatLxBI9gBaebbnrfifHhDYfgasaacH8akY=wiFfYdH8Gipec8Eeeu0xXdbba9frFj0=OqFfea0dXdd9vqai=hGuQ8kuc9pgc9s8qqaq=dirpe0xb9q8qiLsFr0=vr0=vr0dc8meaabaqaciaacaGaaeqabaqabeGadaaakeaacqWG4baEdaWgaaWcbaGaeGymaedabeaakiabg2da9iabdwgaLnaaCaaaleqabaGaeyOeI0IaeqiTdq2aaSbaaWqaaiabigdaXaqabaWccqaHepaDaaGcdaWdbaqaamaacmqabaWaaSaaaeaacqaHYoGydaWgaaWcbaGaeGymaedabeaakiabdIha4naaBaaaleaacqaI1aqnaeqaaOGaeiikaGIaeqiXdq3aaSbaaSqaaiabicdaWaqabaGccqGGPaqkcqGHCeIWcqWGLbqzdaahaaWcbeqaaiabes7aKnaaBaaameaacqaIXaqmaeqaaSGaeqiXdqhaaaGcbaGaemiEaG3aaSbaaSqaaiabiwda1aqabaGccqGGOaakcqaHepaDdaWgaaWcbaGaeGimaadabeaakiabcMcaPiabgUcaRiabcUfaBjabgYriclabgkHiTiabdIha4naaBaaaleaacqaI1aqnaeqaaOGaeiikaGIaeqiXdq3aaSbaaSqaaiabicdaWaqabaGccqGGPaqkcqWGLbqzdaahaaWcbeqaaiabgkHiTiabek7aInaaBaaameaacqaI1aqnaeqaaSGaeqiXdqhaaaaaaOGaay5Eaiaaw2haaaWcbeqab0Gaey4kIipakiabdsgaKjabes8a0jabgUcaRiabdoeadnaaCaaaleqabaGaemitaW0aaSbaaWqaaiabdchaWbqabaaaaOGaemyzau2aaWbaaSqabeaacqGHsislcqaH0oazdaWgaaadbaGaeGymaedabeaaliabes8a0baakiaaxMaacaWLjaWaaiWabeaacqaI3aWnaiaawUhacaGL9baaaaa@7AEA@

and

x4=e−δ4τ∫{β4x5(τ0)ℜeδ4τx5(τ0)+[ℜ−x5(τ0)]e−β5τ}dτ+CRae−δ4τ     {8}
 MathType@MTEF@5@5@+=feaafiart1ev1aaatCvAUfKttLearuWrP9MDH5MBPbIqV92AaeXatLxBI9gBaebbnrfifHhDYfgasaacH8akY=wiFfYdH8Gipec8Eeeu0xXdbba9frFj0=OqFfea0dXdd9vqai=hGuQ8kuc9pgc9s8qqaq=dirpe0xb9q8qiLsFr0=vr0=vr0dc8meaabaqaciaacaGaaeqabaqabeGadaaakeaacqWG4baEdaWgaaWcbaGaeGinaqdabeaakiabg2da9iabdwgaLnaaCaaaleqabaGaeyOeI0IaeqiTdq2aaSbaaWqaaiabisda0aqabaWccqaHepaDaaGcdaWdbaqaamaacmqabaWaaSaaaeaacqaHYoGydaWgaaWcbaGaeGinaqdabeaakiabdIha4naaBaaaleaacqaI1aqnaeqaaOGaeiikaGIaeqiXdq3aaSbaaSqaaiabicdaWaqabaGccqGGPaqkcqGHCeIWcqWGLbqzdaahaaWcbeqaaiabes7aKnaaBaaameaacqaI0aanaeqaaSGaeqiXdqhaaaGcbaGaemiEaG3aaSbaaSqaaiabiwda1aqabaGccqGGOaakcqaHepaDdaWgaaWcbaGaeGimaadabeaakiabcMcaPiabgUcaRiabcUfaBjabgYriclabgkHiTiabdIha4naaBaaaleaacqaI1aqnaeqaaOGaeiikaGIaeqiXdq3aaSbaaSqaaiabicdaWaqabaGccqGGPaqkcqGGDbqxcqWGLbqzdaahaaWcbeqaaiabgkHiTiabek7aInaaBaaameaacqaI1aqnaeqaaSGaeqiXdqhaaaaaaOGaay5Eaiaaw2haaaWcbeqab0Gaey4kIipakiabdsgaKjabes8a0jabgUcaRiabdoeadnaaCaaaleqabaGaemOuai1aaSbaaWqaaiabdggaHbqabaaaaOGaemyzau2aaWbaaSqabeaacqGHsislcqaH0oazdaWgaaadbaGaeGinaqdabeaaliabes8a0baakiaaxMaacaWLjaWaaiWabeaacqaI4aaoaiaawUhacaGL9baaaaa@7C3A@

Also, the time dependent solutions of *L*_*a *_and *R*_*p *_in dimensionless form are given by

x2=e−δ2τ∫β2[x5(τ0)+{ℜ−x5(τ)}e−β5τ]n2{x5(τ0)ℜ}n2+[x5(τ0)+{ℜ−x5(τ0)}e−β5τ]n2dτ+CLae−δ2τ     {9}
 MathType@MTEF@5@5@+=feaafiart1ev1aaatCvAUfKttLearuWrP9MDH5MBPbIqV92AaeXatLxBI9gBaebbnrfifHhDYfgasaacH8akY=wiFfYdH8Gipec8Eeeu0xXdbba9frFj0=OqFfea0dXdd9vqai=hGuQ8kuc9pgc9s8qqaq=dirpe0xb9q8qiLsFr0=vr0=vr0dc8meaabaqaciaacaGaaeqabaqabeGadaaakeaacqWG4baEdaWgaaWcbaGaeGOmaidabeaakiabg2da9iabdwgaLnaaCaaaleqabaGaeyOeI0IaeqiTdq2aaSbaaWqaaiabikdaYaqabaWccqaHepaDaaGcdaWdbaqaamaalaaabaGaeqOSdi2aaSbaaSqaaiabikdaYaqabaGcdaWadiqaaiabdIha4naaBaaaleaacqaI1aqnaeqaaOGaeiikaGIaeqiXdq3aaSbaaSqaaiabicdaWaqabaGccqGGPaqkcqGHRaWkdaGadeqaaiabgYriclabgkHiTiabdIha4naaBaaaleaacqaI1aqnaeqaaOGaeiikaGIaeqiXdqNaeiykaKcacaGL7bGaayzFaaGaemyzau2aaWbaaSqabeaacqGHsislcqaHYoGydaWgaaadbaGaeGynaudabeaaliabes8a0baaaOGaay5waiaaw2faamaaCaaaleqabaGaemOBa42aaSbaaWqaaiabikdaYaqabaaaaaGcbaWaaiWabeaacqWG4baEdaWgaaWcbaGaeGynaudabeaakiabcIcaOiabes8a0naaBaaaleaacqaIWaamaeqaaOGaeiykaKIaeyihHimacaGL7bGaayzFaaWaaWbaaSqabeaacqWGUbGBdaWgaaadbaGaeGOmaidabeaaaaGccqGHRaWkdaWadiqaaiabdIha4naaBaaaleaacqaI1aqnaeqaaOGaeiikaGIaeqiXdq3aaSbaaSqaaiabicdaWaqabaGccqGGPaqkcqGHRaWkdaGadeqaaiabgYriclabgkHiTiabdIha4naaBaaaleaacqaI1aqnaeqaaOGaeiikaGIaeqiXdq3aaSbaaSqaaiabicdaWaqabaGccqGGPaqkaiaawUhacaGL9baacqWGLbqzdaahaaWcbeqaaiabgkHiTiabek7aInaaBaaameaacqaI1aqnaeqaaSGaeqiXdqhaaaGccaGLBbGaayzxaaWaaWbaaSqabeaacqWGUbGBdaWgaaadbaGaeGOmaidabeaaaaaaaaWcbeqab0Gaey4kIipakiabdsgaKjabes8a0jabgUcaRiabdoeadnaaCaaaleqabaGaemitaW0aaSbaaWqaaiabdggaHbqabaaaaOGaemyzau2aaWbaaSqabeaacqGHsislcqaH0oazdaWgaaadbaGaeGOmaidabeaaliabes8a0baakiaaxMaacaWLjaWaaiWabeaacqaI5aqoaiaawUhacaGL9baaaaa@9C9A@

x3=e−δ3τ∫β3[x5(τ0)+{ℜ−x5(τ)}e−β5τ]n3{x5(τ0)ℜ}n3+[x5(τ0)+{ℜ−x5(τ0)}e−β5τ]n3dτ+CRpe−δ3τ     {10}
 MathType@MTEF@5@5@+=feaafiart1ev1aaatCvAUfKttLearuWrP9MDH5MBPbIqV92AaeXatLxBI9gBaebbnrfifHhDYfgasaacH8akY=wiFfYdH8Gipec8Eeeu0xXdbba9frFj0=OqFfea0dXdd9vqai=hGuQ8kuc9pgc9s8qqaq=dirpe0xb9q8qiLsFr0=vr0=vr0dc8meaabaqaciaacaGaaeqabaqabeGadaaakeaacqWG4baEdaWgaaWcbaGaeG4mamdabeaakiabg2da9iabdwgaLnaaCaaaleqabaGaeyOeI0IaeqiTdq2aaSbaaWqaaiabiodaZaqabaWccqaHepaDaaGcdaWdbaqaamaalaaabaGaeqOSdi2aaSbaaSqaaiabiodaZaqabaGcdaWadiqaaiabdIha4naaBaaaleaacqaI1aqnaeqaaOGaeiikaGIaeqiXdq3aaSbaaSqaaiabicdaWaqabaGccqGGPaqkcqGHRaWkdaGadeqaaiabgYriclabgkHiTiabdIha4naaBaaaleaacqaI1aqnaeqaaOGaeiikaGIaeqiXdqNaeiykaKcacaGL7bGaayzFaaGaemyzau2aaWbaaSqabeaacqGHsislcqaHYoGydaWgaaadbaGaeGynaudabeaaliabes8a0baaaOGaay5waiaaw2faamaaCaaaleqabaGaemOBa42aaSbaaWqaaiabiodaZaqabaaaaaGcbaWaaiWabeaacqWG4baEdaWgaaWcbaGaeGynaudabeaakiabcIcaOiabes8a0naaBaaaleaacqaIWaamaeqaaOGaeiykaKIaeyihHimacaGL7bGaayzFaaWaaWbaaSqabeaacqWGUbGBdaWgaaadbaGaeG4mamdabeaaaaGccqGHRaWkdaWadiqaaiabdIha4naaBaaaleaacqaI1aqnaeqaaOGaeiikaGIaeqiXdq3aaSbaaSqaaiabicdaWaqabaGccqGGPaqkcqGHRaWkdaGadeqaaiabgYriclabgkHiTiabdIha4naaBaaaleaacqaI1aqnaeqaaOGaeiikaGIaeqiXdq3aaSbaaSqaaiabicdaWaqabaGccqGGPaqkaiaawUhacaGL9baacqWGLbqzdaahaaWcbeqaaiabgkHiTiabek7aInaaBaaameaacqaI1aqnaeqaaSGaeqiXdqhaaaGccaGLBbGaayzxaaWaaWbaaSqabeaacqWGUbGBdaWgaaadbaGaeG4mamdabeaaaaaaaaWcbeqab0Gaey4kIipakiabdsgaKjabes8a0jabgUcaRiabdoeadnaaCaaaleqabaGaemOuai1aaSbaaWqaaiabdchaWbqabaaaaOGaemyzau2aaWbaaSqabeaacqGHsislcqaH0oazdaWgaaadbaGaeG4mamdabeaaliabes8a0baakiaaxMaacaWLjaWaaiWabeaacqaIXaqmcqaIWaamaiaawUhacaGL9baaaaa@9DB0@

Where CLp,CLa,CRa
 MathType@MTEF@5@5@+=feaafiart1ev1aaatCvAUfKttLearuWrP9MDH5MBPbIqV92AaeXatLxBI9gBaebbnrfifHhDYfgasaacH8akY=wiFfYdH8Gipec8Eeeu0xXdbba9frFj0=OqFfea0dXdd9vqai=hGuQ8kuc9pgc9s8qqaq=dirpe0xb9q8qiLsFr0=vr0=vr0dc8meaabaqaciaacaGaaeqabaqabeGadaaakeaacqWGdbWqdaahaaWcbeqaaiabdYeamnaaBaaameaacqWGWbaCaeqaaaaakiabcYcaSiabdoeadnaaCaaaleqabaGaemitaW0aaSbaaWqaaiabdggaHbqabaaaaOGaeiilaWIaem4qam0aaWbaaSqabeaacqWGsbGudaWgaaadbaGaemyyaegabeaaaaaaaa@3A29@ and CRp
 MathType@MTEF@5@5@+=feaafiart1ev1aaatCvAUfKttLearuWrP9MDH5MBPbIqV92AaeXatLxBI9gBaebbnrfifHhDYfgasaacH8akY=wiFfYdH8Gipec8Eeeu0xXdbba9frFj0=OqFfea0dXdd9vqai=hGuQ8kuc9pgc9s8qqaq=dirpe0xb9q8qiLsFr0=vr0=vr0dc8meaabaqaciaacaGaaeqabaqabeGadaaakeaacqWGdbWqdaahaaWcbeqaaiabdkfasnaaBaaameaacqWGWbaCaeqaaaaaaaa@30AB@ are the constants of integration, which can be obtained from the initial condition τ = τ_0_.

**Figure 4:**
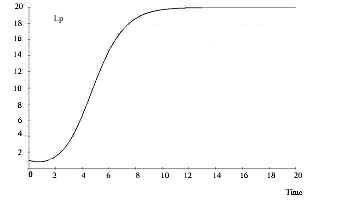
Stress induced Lp growth curve with respect to time (in dimensionless form).

**Figure 5:**
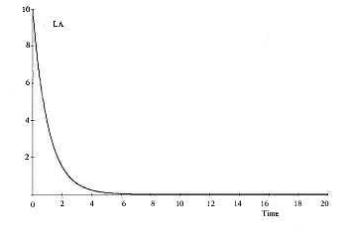
Stress induced La growth curve with respect to time (in dimensionless form).

A detailed numerical solution is shown graphically in Figures 4 and 5 and the values of the parameters are given Table 2. The MATHCAD 13 computer software was used to obtain these numerical solutions.

**Table 2:** The ranges of all the parameters used in our equations

**Parameter**	**Range of numerical values**
α_1_	0.68 ≥ α_1 _≥ 0.068
α_2_	1.43 ≥ α_2 _≥ 0.143
α_3_	1.43 ≥ α_3 _≥ 0.143
α_4_	0.68 ≥ α_4 _≥ 0.068
α_5_	0.16 ≥ α_5 _≥ 0.016
γ_1_	0.122 ≥ γ_1 _≥ 1.222 × 10^-3^
γ_2_	0.014 ≥ γ_2 _≥ 1.422 × 10^-4^
γ_3_	0.014 ≥ γ_3 _≥ 1.422 × 10^-4^
γ_4_	0.122 ≥ γ_4 _≥ 1.222 × 10^-3^
γ_5_	16.4 ≥ γ_5 _≥ 0.016
*n*_1_	*n*_1 _= 1.0
*n*_2_	*n*_2 _= 1.0
*n*_3_	*n*_3 _= 1.0
*n*_4_	*n*_4 _= 1.0
*h*	0.1 ≤ *h *≤ 1.0

To solve system {3} we used the Romberg method of Integration with TOL (tolerance) to the order of 10 ^-3^.

The computer-simulated outcomes of model-1 are depicted in Figures 4 and 5. The *R*_*a *_and *L*_*p *_growth curves show similar outcomes. The *L*_*a *_and *R*_*p *_growth curves are also analogous.

The outcomes of this model show that *L*_*p *_concentration heads towards a saturation point (carrying capacity), whereas *L*_*a *_concentration gradually diminishes. This indicates that stress alone can lead the brain to a catastrophic state in which depression may become uncontrollable. An unpredictable event may arise beyond this catastrophic point (maximum sustainable carrying capacity). It also shows the imbalance and dynamically opposite characteristics implicit in the lateral hemispheric division of the brain. However, model-1 does not consider the effects of exercise and stress together; that is incorporated in model-2.

### Model-2: The effects of concomitant stress and exercise on the four different quadrants of the brain

**Figure 6:**
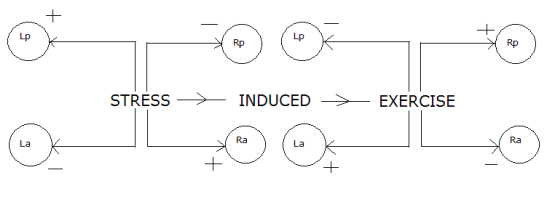
Schematic diagram of stress-induced exercise activity within the brain.

As a non-pharmacological intervention, we have introduced 'exercise' into the stress dynamics. The schematic diagram shown in Figure 6 represents the functional characteristics of brain dynamics in presence of stress-induced exercise activities. In this particular schema we assume that both stress and exercise are acting simultaneously where the stress activity (not counting "moderate" exercise itself as a stressor, whereas "heavy" exercise may qualify as a stressor) develops independently from various sources and/or systems over which the individual has no control.

A person who is not under the influence of stress can do exercise. On the other hand one can do the exercise when one knows that one is under influence of stress. We call this situation 'stress-induced exercise activity'. In the present study, our approach is based on the latter scenario.

In this scenario, the effects of exercise positively activate the left-anterior and right-posterior of the brain but they negatively activate (feedback mechanism) the left-posterior and right anterior of the brain. As such, the exercise effect conteracts the stress effect on the brain.

Based on the above schematic diagram we have developed the following mathematical model.

Model-2 (Figure 6) may be defined as:

ddt(Lp)=α1(St)hn1+(Ex)n1−γ1(Lp)ddt(La)=α2(Ex)hn2+(St)n2−γ2(La)ddt(Rp)=α3(Ex)hn3+(St)n3−γ3(Rp)ddt(Ra)=α4(St)hn4+(Ex)n4−γ4(Ra)ddt(St)=α5(St)−γ5(St)(Ex)ddt(Ex)=α6(St)(Ex)−γ6(Ex)     {11}
 MathType@MTEF@5@5@+=feaafiart1ev1aaatCvAUfKttLearuWrP9MDH5MBPbIqV92AaeXatLxBI9gBaebbnrfifHhDYfgasaacH8akY=wiFfYdH8Gipec8Eeeu0xXdbba9frFj0=OqFfea0dXdd9vqai=hGuQ8kuc9pgc9s8qqaq=dirpe0xb9q8qiLsFr0=vr0=vr0dc8meaabaqaciaacaGaaeqabaqabeGadaaakeaafaqaaeGbbaaaaeaadaWcaaqaaiabdsgaKbqaaiabdsgaKjabdsha0baacqGGOaakcqWGmbatcqWGWbaCcqGGPaqkcqGH9aqpdaWcaaqaaiabeg7aHnaaBaaaleaacqaIXaqmaeqaaOGaeiikaGIaem4uamLaemiDaqNaeiykaKcabaGaemiAaG2aaWbaaSqabeaacqWGUbGBdaWgaaadbaGaeGymaedabeaaaaGccqGHRaWkcqGGOaakcqWGfbqrcqWG4baEcqGGPaqkdaahaaWcbeqaaiabd6gaUnaaBaaameaacqaIXaqmaeqaaaaaaaGccqGHsislcqaHZoWzdaWgaaWcbaGaeGymaedabeaakiabcIcaOiabdYeamjabdchaWjabcMcaPaqaamaalaaabaGaemizaqgabaGaemizaqMaemiDaqhaaiabcIcaOiabdYeamjabdggaHjabcMcaPiabg2da9maalaaabaGaeqySde2aaSbaaSqaaiabikdaYaqabaGccqGGOaakcqWGfbqrcqWG4baEcqGGPaqkaeaacqWGObaAdaahaaWcbeqaaiabd6gaUnaaBaaameaacqaIYaGmaeqaaaaakiabgUcaRiabcIcaOiabdofatjabdsha0jabcMcaPmaaCaaaleqabaGaemOBa42aaSbaaWqaaiabikdaYaqabaaaaaaakiabgkHiTiabeo7aNnaaBaaaleaacqaIYaGmaeqaaOGaeiikaGIaemitaWKaemyyaeMaeiykaKcabaWaaSaaaeaacqWGKbazaeaacqWGKbazcqWG0baDaaGaeiikaGIaemOuaiLaemiCaaNaeiykaKIaeyypa0ZaaSaaaeaacqaHXoqydaWgaaWcbaGaeG4mamdabeaakiabcIcaOiabdweafjabdIha4jabcMcaPaqaaiabdIgaOnaaCaaaleqabaGaemOBa42aaSbaaWqaaiabiodaZaqabaaaaOGaey4kaSIaeiikaGIaem4uamLaemiDaqNaeiykaKYaaWbaaSqabeaacqWGUbGBdaWgaaadbaGaeG4mamdabeaaaaaaaOGaeyOeI0Iaeq4SdC2aaSbaaSqaaiabiodaZaqabaGccqGGOaakcqWGsbGucqWGWbaCcqGGPaqkaeaadaWcaaqaaiabdsgaKbqaaiabdsgaKjabdsha0baacqGGOaakcqWGsbGucqWGHbqycqGGPaqkcqGH9aqpdaWcaaqaaiabeg7aHnaaBaaaleaacqaI0aanaeqaaOGaeiikaGIaem4uamLaemiDaqNaeiykaKcabaGaemiAaG2aaWbaaSqabeaacqWGUbGBdaWgaaadbaGaeGinaqdabeaaaaGccqGHRaWkcqGGOaakcqWGfbqrcqWG4baEcqGGPaqkdaahaaWcbeqaaiabd6gaUnaaBaaameaacqaI0aanaeqaaaaaaaGccqGHsislcqaHZoWzdaWgaaWcbaGaeGinaqdabeaakiabcIcaOiabdkfasjabdggaHjabcMcaPaqaamaalaaabaGaemizaqgabaGaemizaqMaemiDaqhaaiabcIcaOiabdofatjabdsha0jabcMcaPiabg2da9iabeg7aHnaaBaaaleaacqaI1aqnaeqaaOGaeiikaGIaem4uamLaemiDaqNaeiykaKIaeyOeI0Iaeq4SdC2aaSbaaSqaaiabiwda1aqabaGccqGGOaakcqWGtbWucqWG0baDcqGGPaqkcqGGOaakcqWGfbqrcqWG4baEcqGGPaqkaeaadaWcaaqaaiabdsgaKbqaaiabdsgaKjabdsha0baacqGGOaakcqWGfbqrcqWG4baEcqGGPaqkcqGH9aqpcqaHXoqydaWgaaWcbaGaeGOnaydabeaakiabcIcaOiabdofatjabdsha0jabcMcaPiabcIcaOiabdweafjabdIha4jabcMcaPiabgkHiTiabeo7aNnaaBaaaleaacqaI2aGnaeqaaOGaeiikaGIaemyrauKaemiEaGNaeiykaKcaaiaaxMaacaWLjaWaaiWabeaacqaIXaqmcqaIXaqmaiaawUhacaGL9baaaaa@FD66@

Where (*Ex*) denotes the exercise activity and *n*_1_, *n*_4 _are Hill coefficients; α_6 _is the exercise generation due to stress, γ_5 _is the degradation of stress due to exercise and γ_6 _is the degradation of exercise effects.

The non-trivial steady state of the above system is as follows:

Lp0=(γ1α1)[(St)hn1+(Ex)n1]>0,La0=(γ2α2)[(Ex)hn2+(St)n2]>0Rp0=(γ3α3)[(Ex)hn3+(St)n3]>0,Ra0=(γ4α4)[(St)hn4+(Ex)n4]>0St0=γ6α6>0,Ex0=α5γ5>0     {12}
 MathType@MTEF@5@5@+=feaafiart1ev1aaatCvAUfKttLearuWrP9MDH5MBPbIqV92AaeXatLxBI9gBaebbnrfifHhDYfgasaacH8akY=wiFfYdH8Gipec8Eeeu0xXdbba9frFj0=OqFfea0dXdd9vqai=hGuQ8kuc9pgc9s8qqaq=dirpe0xb9q8qiLsFr0=vr0=vr0dc8meaabaqaciaacaGaaeqabaqabeGadaaakeaafaqaaeWabaaabaGaemitaW0aa0baaSqaaiabdchaWbqaaiabicdaWaaakiabg2da9maabmGabaWaaSaaaeaacqaHZoWzdaWgaaWcbaGaeGymaedabeaaaOqaaiabeg7aHnaaBaaaleaacqaIXaqmaeqaaaaaaOGaayjkaiaawMcaamaadmGabaWaaSaaaeaacqGGOaakcqWGtbWucqWG0baDcqGGPaqkaeaacqWGObaAdaahaaWcbeqaaiabd6gaUnaaBaaameaacqaIXaqmaeqaaaaakiabgUcaRiabcIcaOiabdweafjabdIha4jabcMcaPmaaCaaaleqabaGaemOBa42aaSbaaWqaaiabigdaXaqabaaaaaaaaOGaay5waiaaw2faaiabg6da+iabicdaWiabcYcaSiabdYeamnaaDaaaleaacqWGHbqyaeaacqaIWaamaaGccqGH9aqpdaqadiqaamaalaaabaGaeq4SdC2aaSbaaSqaaiabikdaYaqabaaakeaacqaHXoqydaWgaaWcbaGaeGOmaidabeaaaaaakiaawIcacaGLPaaadaWadiqaamaalaaabaGaeiikaGIaemyrauKaemiEaGNaeiykaKcabaGaemiAaG2aaWbaaSqabeaacqWGUbGBdaWgaaadbaGaeGOmaidabeaaaaGccqGHRaWkcqGGOaakcqWGtbWucqWG0baDcqGGPaqkdaahaaWcbeqaaiabd6gaUnaaBaaameaacqaIYaGmaeqaaaaaaaaakiaawUfacaGLDbaacqGH+aGpcqaIWaamaeaacqWGsbGudaqhaaWcbaGaemiCaahabaGaeGimaadaaOGaeyypa0ZaaeWaceaadaWcaaqaaiabeo7aNnaaBaaaleaacqaIZaWmaeqaaaGcbaGaeqySde2aaSbaaSqaaiabiodaZaqabaaaaaGccaGLOaGaayzkaaWaamWaceaadaWcaaqaaiabcIcaOiabdweafjabdIha4jabcMcaPaqaaiabdIgaOnaaCaaaleqabaGaemOBa42aaSbaaWqaaiabiodaZaqabaaaaOGaey4kaSIaeiikaGIaem4uamLaemiDaqNaeiykaKYaaWbaaSqabeaacqWGUbGBdaWgaaadbaGaeG4mamdabeaaaaaaaaGccaGLBbGaayzxaaGaeyOpa4JaeGimaaJaeiilaWIaemOuai1aa0baaSqaaiabdggaHbqaaiabicdaWaaakiabg2da9maabmGabaWaaSaaaeaacqaHZoWzdaWgaaWcbaGaeGinaqdabeaaaOqaaiabeg7aHnaaBaaaleaacqaI0aanaeqaaaaaaOGaayjkaiaawMcaamaadmGabaWaaSaaaeaacqGGOaakcqWGtbWucqWG0baDcqGGPaqkaeaacqWGObaAdaahaaWcbeqaaiabd6gaUnaaBaaameaacqaI0aanaeqaaaaakiabgUcaRiabcIcaOiabdweafjabdIha4jabcMcaPmaaCaaaleqabaGaemOBa42aaSbaaWqaaiabisda0aqabaaaaaaaaOGaay5waiaaw2faaiabg6da+iabicdaWaqaaiabdofatjabdsha0naaCaaaleqabaGaeGimaadaaOGaeyypa0ZaaSaaaeaacqaHZoWzdaWgaaWcbaGaeGOnaydabeaaaOqaaiabeg7aHnaaBaaaleaacqaI2aGnaeqaaaaakiabg6da+iabicdaWiabcYcaSiabdweafjabdIha4naaCaaaleqabaGaeGimaadaaOGaeyypa0ZaaSaaaeaacqaHXoqydaWgaaWcbaGaeGynaudabeaaaOqaaiabeo7aNnaaBaaaleaacqaI1aqnaeqaaaaakiabg6da+iabicdaWaaacaWLjaGaaCzcamaacmqabaGaeGymaeJaeGOmaidacaGL7bGaayzFaaaaaa@CDF8@

### Steady state and linearization

The dimensionless form of Eq. {11} is:

dx1dτ=ξ1x51+x6n1−ζ1x1dx2dτ=ξ2x61+x5n2−ζ2x2dx3dτ=ξ3x61+x5n3−ζ3x3dx4dτ=ξ4x51+x6n4−ζ4x4dx5dτ=ξ5x5−ζ5x5x6dx6dτ=ξ6x5x6−ζ6x6     {13}
 MathType@MTEF@5@5@+=feaafiart1ev1aaatCvAUfKttLearuWrP9MDH5MBPbIqV92AaeXatLxBI9gBaebbnrfifHhDYfgasaacH8akY=wiFfYdH8Gipec8Eeeu0xXdbba9frFj0=OqFfea0dXdd9vqai=hGuQ8kuc9pgc9s8qqaq=dirpe0xb9q8qiLsFr0=vr0=vr0dc8meaabaqaciaacaGaaeqabaqabeGadaaakeaafaqaaeGbbaaaaeaadaWcaaqaaiabdsgaKjabdIha4naaBaaaleaacqaIXaqmaeqaaaGcbaGaemizaqMaeqiXdqhaaiabg2da9maalaaabaGaeqOVdG3aaSbaaSqaaiabigdaXaqabaGccqWG4baEdaWgaaWcbaGaeGynaudabeaaaOqaaiabigdaXiabgUcaRiabdIha4naaDaaaleaacqaI2aGnaeaacqWGUbGBdaWgaaadbaGaeGymaedabeaaaaaaaOGaeyOeI0IaeqOTdO3aaSbaaSqaaiabigdaXaqabaGccqWG4baEdaWgaaWcbaGaeGymaedabeaaaOqaamaalaaabaGaemizaqMaemiEaG3aaSbaaSqaaiabikdaYaqabaaakeaacqWGKbazcqaHepaDaaGaeyypa0ZaaSaaaeaacqaH+oaEdaWgaaWcbaGaeGOmaidabeaakiabdIha4naaBaaaleaacqaI2aGnaeqaaaGcbaGaeGymaeJaey4kaSIaemiEaG3aa0baaSqaaiabiwda1aqaaiabd6gaUnaaBaaameaacqaIYaGmaeqaaaaaaaGccqGHsislcqaH2oGEdaWgaaWcbaGaeGOmaidabeaakiabdIha4naaBaaaleaacqaIYaGmaeqaaaGcbaWaaSaaaeaacqWGKbazcqWG4baEdaWgaaWcbaGaeG4mamdabeaaaOqaaiabdsgaKjabes8a0baacqGH9aqpdaWcaaqaaiabe67a4naaBaaaleaacqaIZaWmaeqaaOGaemiEaG3aaSbaaSqaaiabiAda2aqabaaakeaacqaIXaqmcqGHRaWkcqWG4baEdaqhaaWcbaGaeGynaudabaGaemOBa42aaSbaaWqaaiabiodaZaqabaaaaaaakiabgkHiTiabeA7a6naaBaaaleaacqaIZaWmaeqaaOGaemiEaG3aaSbaaSqaaiabiodaZaqabaaakeaadaWcaaqaaiabdsgaKjabdIha4naaBaaaleaacqaI0aanaeqaaaGcbaGaemizaqMaeqiXdqhaaiabg2da9maalaaabaGaeqOVdG3aaSbaaSqaaiabisda0aqabaGccqWG4baEdaWgaaWcbaGaeGynaudabeaaaOqaaiabigdaXiabgUcaRiabdIha4naaDaaaleaacqaI2aGnaeaacqWGUbGBdaWgaaadbaGaeGinaqdabeaaaaaaaOGaeyOeI0IaeqOTdO3aaSbaaSqaaiabisda0aqabaGccqWG4baEdaWgaaWcbaGaeGinaqdabeaaaOqaamaalaaabaGaemizaqMaemiEaG3aaSbaaSqaaiabiwda1aqabaaakeaacqWGKbazcqaHepaDaaGaeyypa0JaeqOVdG3aaSbaaSqaaiabiwda1aqabaGccqWG4baEdaWgaaWcbaGaeGynaudabeaakiabgkHiTiabeA7a6naaBaaaleaacqaI1aqnaeqaaOGaemiEaG3aaSbaaSqaaiabiwda1aqabaGccqWG4baEdaWgaaWcbaGaeGOnaydabeaaaOqaamaalaaabaGaemizaqMaemiEaG3aaSbaaSqaaiabiAda2aqabaaakeaacqWGKbazcqaHepaDaaGaeyypa0JaeqOVdG3aaSbaaSqaaiabiAda2aqabaGccqWG4baEdaWgaaWcbaGaeGynaudabeaakiabdIha4naaBaaaleaacqaI2aGnaeqaaOGaeyOeI0IaeqOTdO3aaSbaaSqaaiabiAda2aqabaGccqWG4baEdaWgaaWcbaGaeGOnaydabeaaaaGccaWLjaGaaCzcamaacmqabaGaeGymaeJaeG4mamdacaGL7bGaayzFaaaaaa@CCCA@

Where



Let (x10,x20,x30,x40,x50,x60
 MathType@MTEF@5@5@+=feaafiart1ev1aaatCvAUfKttLearuWrP9MDH5MBPbIqV92AaeXatLxBI9gBaebbnrfifHhDYfgasaacH8akY=wiFfYdH8Gipec8Eeeu0xXdbba9frFj0=OqFfea0dXdd9vqai=hGuQ8kuc9pgc9s8qqaq=dirpe0xb9q8qiLsFr0=vr0=vr0dc8meaabaqaciaacaGaaeqabaqabeGadaaakeaacqWG4baEdaqhaaWcbaGaeGymaedabaGaeGimaadaaOGaeiilaWIaemiEaG3aa0baaSqaaiabikdaYaqaaiabicdaWaaakiabcYcaSiabdIha4naaDaaaleaacqaIZaWmaeaacqaIWaamaaGccqGGSaalcqWG4baEdaqhaaWcbaGaeGinaqdabaGaeGimaadaaOGaeiilaWIaemiEaG3aa0baaSqaaiabiwda1aqaaiabicdaWaaakiabcYcaSiabdIha4naaDaaaleaacqaI2aGnaeaacqaIWaamaaaaaa@4674@) be the dimensionless steady state values; then for *u*_*i *_= *x*_*i *_- xi0
 MathType@MTEF@5@5@+=feaafiart1ev1aaatCvAUfKttLearuWrP9MDH5MBPbIqV92AaeXatLxBI9gBaebbnrfifHhDYfgasaacH8akY=wiFfYdH8Gipec8Eeeu0xXdbba9frFj0=OqFfea0dXdd9vqai=hGuQ8kuc9pgc9s8qqaq=dirpe0xb9q8qiLsFr0=vr0=vr0dc8meaabaqaciaacaGaaeqabaqabeGadaaakeaacqWG4baEdaqhaaWcbaGaemyAaKgabaGaeGimaadaaaaa@309B@ (*i *= 1,.......,6) the linearization version of the above system is:

du1dτ=−ζ1u1+(ζ1x50)u5−ζ12n1(x60)n1−1(x10)2ξ1x50u6du2dτ=−ζ2u2−ζ22n2(x50)n2−1(x20)2ξ2x60u5+(ζ2x60)u6du3dτ=−ζ3u3−ζ32n3(x50)n3−1(x30)2ξ3x60u5+(ζ3x60)u6du4dτ=−ζ4u4+(ζ4x50)u5−ζ42n4(x60)n4−1(x40)2ξ4x50u6du5dτ=−ζ5x50u6du6dτ=−ξ6x60u5     {15}
 MathType@MTEF@5@5@+=feaafiart1ev1aaatCvAUfKttLearuWrP9MDH5MBPbIqV92AaeXatLxBI9gBaebbnrfifHhDYfgasaacH8akY=wiFfYdH8Gipec8Eeeu0xXdbba9frFj0=OqFfea0dXdd9vqai=hGuQ8kuc9pgc9s8qqaq=dirpe0xb9q8qiLsFr0=vr0=vr0dc8meaabaqaciaacaGaaeqabaqabeGadaaakeaafaqaaeGbbaaaaeaadaWcaaqaaiabdsgaKjabdwha1naaBaaaleaacqaIXaqmaeqaaaGcbaGaemizaqMaeqiXdqhaaiabg2da9iabgkHiTiabeA7a6naaBaaaleaacqaIXaqmaeqaaOGaemyDau3aaSbaaSqaaiabigdaXaqabaGccqGHRaWkdaqadiqaamaalaaabaGaeqOTdO3aaSbaaSqaaiabigdaXaqabaaakeaacqWG4baEdaqhaaWcbaGaeGynaudabaGaeGimaadaaaaaaOGaayjkaiaawMcaaiabdwha1naaBaaaleaacqaI1aqnaeqaaOGaeyOeI0YaaSaaaeaacqaH2oGEdaqhaaWcbaGaeGymaedabaGaeGOmaidaaOGaemOBa42aaSbaaSqaaiabigdaXaqabaGccqGGOaakcqWG4baEdaqhaaWcbaGaeGOnaydabaGaeGimaadaaOGaeiykaKYaaWbaaSqabeaacqWGUbGBdaWgaaadbaGaeGymaedabeaaliabgkHiTiabigdaXaaakiabcIcaOiabdIha4naaDaaaleaacqaIXaqmaeaacqaIWaamaaGccqGGPaqkdaahaaWcbeqaaiabikdaYaaaaOqaaiabe67a4naaBaaaleaacqaIXaqmaeqaaOGaemiEaG3aa0baaSqaaiabiwda1aqaaiabicdaWaaaaaGccqWG1bqDdaWgaaWcbaGaeGOnaydabeaaaOqaamaalaaabaGaemizaqMaemyDau3aaSbaaSqaaiabikdaYaqabaaakeaacqWGKbazcqaHepaDaaGaeyypa0JaeyOeI0IaeqOTdO3aaSbaaSqaaiabikdaYaqabaGccqWG1bqDdaWgaaWcbaGaeGOmaidabeaakiabgkHiTmaalaaabaGaeqOTdO3aa0baaSqaaiabikdaYaqaaiabikdaYaaakiabd6gaUnaaBaaaleaacqaIYaGmaeqaaOGaeiikaGIaemiEaG3aa0baaSqaaiabiwda1aqaaiabicdaWaaakiabcMcaPmaaCaaaleqabaGaemOBa42aaSbaaWqaaiabikdaYaqabaWccqGHsislcqaIXaqmaaGccqGGOaakcqWG4baEdaqhaaWcbaGaeGOmaidabaGaeGimaadaaOGaeiykaKYaaWbaaSqabeaacqaIYaGmaaaakeaacqaH+oaEdaWgaaWcbaGaeGOmaidabeaakiabdIha4naaDaaaleaacqaI2aGnaeaacqaIWaamaaaaaOGaemyDau3aaSbaaSqaaiabiwda1aqabaGccqGHRaWkdaqadiqaamaalaaabaGaeqOTdO3aaSbaaSqaaiabikdaYaqabaaakeaacqWG4baEdaqhaaWcbaGaeGOnaydabaGaeGimaadaaaaaaOGaayjkaiaawMcaaiabdwha1naaBaaaleaacqaI2aGnaeqaaaGcbaWaaSaaaeaacqWGKbazcqWG1bqDdaWgaaWcbaGaeG4mamdabeaaaOqaaiabdsgaKjabes8a0baacqGH9aqpcqGHsislcqaH2oGEdaWgaaWcbaGaeG4mamdabeaakiabdwha1naaBaaaleaacqaIZaWmaeqaaOGaeyOeI0YaaSaaaeaacqaH2oGEdaqhaaWcbaGaeG4mamdabaGaeGOmaidaaOGaemOBa42aaSbaaSqaaiabiodaZaqabaGccqGGOaakcqWG4baEdaqhaaWcbaGaeGynaudabaGaeGimaadaaOGaeiykaKYaaWbaaSqabeaacqWGUbGBdaWgaaadbaGaeG4mamdabeaaliabgkHiTiabigdaXaaakiabcIcaOiabdIha4naaDaaaleaacqaIZaWmaeaacqaIWaamaaGccqGGPaqkdaahaaWcbeqaaiabikdaYaaaaOqaaiabe67a4naaBaaaleaacqaIZaWmaeqaaOGaemiEaG3aa0baaSqaaiabiAda2aqaaiabicdaWaaaaaGccqWG1bqDdaWgaaWcbaGaeGynaudabeaakiabgUcaRmaabmGabaWaaSaaaeaacqaH2oGEdaWgaaWcbaGaeG4mamdabeaaaOqaaiabdIha4naaDaaaleaacqaI2aGnaeaacqaIWaamaaaaaaGccaGLOaGaayzkaaGaemyDau3aaSbaaSqaaiabiAda2aqabaaakeaadaWcaaqaaiabdsgaKjabdwha1naaBaaaleaacqaI0aanaeqaaaGcbaGaemizaqMaeqiXdqhaaiabg2da9iabgkHiTiabeA7a6naaBaaaleaacqaI0aanaeqaaOGaemyDau3aaSbaaSqaaiabisda0aqabaGccqGHRaWkdaqadiqaamaalaaabaGaeqOTdO3aaSbaaSqaaiabisda0aqabaaakeaacqWG4baEdaqhaaWcbaGaeGynaudabaGaeGimaadaaaaaaOGaayjkaiaawMcaaiabdwha1naaBaaaleaacqaI1aqnaeqaaOGaeyOeI0YaaSaaaeaacqaH2oGEdaqhaaWcbaGaeGinaqdabaGaeGOmaidaaOGaemOBa42aaSbaaSqaaiabisda0aqabaGccqGGOaakcqWG4baEdaqhaaWcbaGaeGOnaydabaGaeGimaadaaOGaeiykaKYaaWbaaSqabeaacqWGUbGBdaWgaaadbaGaeGinaqdabeaaliabgkHiTiabigdaXaaakiabcIcaOiabdIha4naaDaaaleaacqaI0aanaeaacqaIWaamaaGccqGGPaqkdaahaaWcbeqaaiabikdaYaaaaOqaaiabe67a4naaBaaaleaacqaI0aanaeqaaOGaemiEaG3aa0baaSqaaiabiwda1aqaaiabicdaWaaaaaGccqWG1bqDdaWgaaWcbaGaeGOnaydabeaaaOqaamaalaaabaGaemizaqMaemyDau3aaSbaaSqaaiabiwda1aqabaaakeaacqWGKbazcqaHepaDaaGaeyypa0JaeyOeI0IaeqOTdO3aaSbaaSqaaiabiwda1aqabaGccqWG4baEdaqhaaWcbaGaeGynaudabaGaeGimaadaaOGaemyDau3aaSbaaSqaaiabiAda2aqabaaakeaadaWcaaqaaiabdsgaKjabdwha1naaBaaaleaacqaI2aGnaeqaaaGcbaGaemizaqMaeqiXdqhaaiabg2da9iabgkHiTiabe67a4naaBaaaleaacqaI2aGnaeqaaOGaemiEaG3aa0baaSqaaiabiAda2aqaaiabicdaWaaakiabdwha1naaBaaaleaacqaI1aqnaeqaaaaakiaaxMaacaWLjaWaaiWabeaacqaIXaqmcqaI1aqnaiaawUhacaGL9baaaaa@429E@

The characteristic equation of the above system is given by:

(ζ1x10+λ)(ζ2x20+λ)(ζ3x30+λ)(ζ4x40+λ)(λ2+ξ6ζ5x50x60)=0     {16}
 MathType@MTEF@5@5@+=feaafiart1ev1aaatCvAUfKttLearuWrP9MDH5MBPbIqV92AaeXatLxBI9gBaebbnrfifHhDYfgasaacH8akY=wiFfYdH8Gipec8Eeeu0xXdbba9frFj0=OqFfea0dXdd9vqai=hGuQ8kuc9pgc9s8qqaq=dirpe0xb9q8qiLsFr0=vr0=vr0dc8meaabaqaciaacaGaaeqabaqabeGadaaakeaadaqadiqaamaalaaabaGaeqOTdO3aaSbaaSqaaiabigdaXaqabaaakeaacqWG4baEdaqhaaWcbaGaeGymaedabaGaeGimaadaaaaakiabgUcaRiabeU7aSbGaayjkaiaawMcaamaabmGabaWaaSaaaeaacqaH2oGEdaWgaaWcbaGaeGOmaidabeaaaOqaaiabdIha4naaDaaaleaacqaIYaGmaeaacqaIWaamaaaaaOGaey4kaSIaeq4UdWgacaGLOaGaayzkaaWaaeWaceaadaWcaaqaaiabeA7a6naaBaaaleaacqaIZaWmaeqaaaGcbaGaemiEaG3aa0baaSqaaiabiodaZaqaaiabicdaWaaaaaGccqGHRaWkcqaH7oaBaiaawIcacaGLPaaadaqadiqaamaalaaabaGaeqOTdO3aaSbaaSqaaiabisda0aqabaaakeaacqWG4baEdaqhaaWcbaGaeGinaqdabaGaeGimaadaaaaakiabgUcaRiabeU7aSbGaayjkaiaawMcaamaabmGabaGaeq4UdW2aaWbaaSqabeaacqaIYaGmaaGccqGHRaWkcqaH+oaEdaWgaaWcbaGaeGOnaydabeaakiabeA7a6naaBaaaleaacqaI1aqnaeqaaOGaemiEaG3aa0baaSqaaiabiwda1aqaaiabicdaWaaakiabdIha4naaDaaaleaacqaI2aGnaeaacqaIWaamaaaakiaawIcacaGLPaaacqGH9aqpcqaIWaamcaWLjaGaaCzcamaacmqabaGaeGymaeJaeGOnaydacaGL7bGaayzFaaaaaa@70F6@

The possible roots of the characteristic equation are:

λ1=−ζ1x10,λ2=−ζ2x20,λ3=−ζ3x30,λ4=−ζ4x40,λ5=iξ6ζ5x50x60,λ6=−iξ6ζ5x50x60     {17}
 MathType@MTEF@5@5@+=feaafiart1ev1aaatCvAUfKttLearuWrP9MDH5MBPbIqV92AaeXatLxBI9gBaebbnrfifHhDYfgasaacH8akY=wiFfYdH8Gipec8Eeeu0xXdbba9frFj0=OqFfea0dXdd9vqai=hGuQ8kuc9pgc9s8qqaq=dirpe0xb9q8qiLsFr0=vr0=vr0dc8meaabaqaciaacaGaaeqabaqabeGadaaakeaacqaH7oaBdaWgaaWcbaGaeGymaedabeaakiabg2da9iabgkHiTmaalaaabaGaeqOTdO3aaSbaaSqaaiabigdaXaqabaaakeaacqWG4baEdaqhaaWcbaGaeGymaedabaGaeGimaadaaaaakiabcYcaSiabeU7aSnaaBaaaleaacqaIYaGmaeqaaOGaeyypa0JaeyOeI0YaaSaaaeaacqaH2oGEdaWgaaWcbaGaeGOmaidabeaaaOqaaiabdIha4naaDaaaleaacqaIYaGmaeaacqaIWaamaaaaaOGaeiilaWIaeq4UdW2aaSbaaSqaaiabiodaZaqabaGccqGH9aqpcqGHsisldaWcaaqaaiabeA7a6naaBaaaleaacqaIZaWmaeqaaaGcbaGaemiEaG3aa0baaSqaaiabiodaZaqaaiabicdaWaaaaaGccqGGSaalcqaH7oaBdaWgaaWcbaGaeGinaqdabeaakiabg2da9iabgkHiTmaalaaabaGaeqOTdO3aaSbaaSqaaiabisda0aqabaaakeaacqWG4baEdaqhaaWcbaGaeGinaqdabaGaeGimaadaaaaakiabcYcaSiabeU7aSnaaBaaaleaacqaI1aqnaeqaaOGaeyypa0JaemyAaK2aaOaaaeaacqaH+oaEdaWgaaWcbaGaeGOnaydabeaakiabeA7a6naaBaaaleaacqaI1aqnaeqaaOGaemiEaG3aa0baaSqaaiabiwda1aqaaiabicdaWaaakiabdIha4naaDaaaleaacqaI2aGnaeaacqaIWaamaaaabeaakiabcYcaSiabeU7aSnaaBaaaleaacqaI2aGnaeqaaOGaeyypa0JaeyOeI0IaemyAaK2aaOaaaeaacqaH+oaEdaWgaaWcbaGaeGOnaydabeaakiabeA7a6naaBaaaleaacqaI1aqnaeqaaOGaemiEaG3aa0baaSqaaiabiwda1aqaaiabicdaWaaakiabdIha4naaDaaaleaacqaI2aGnaeaacqaIWaamaaaabeaakiaaxMaacaWLjaWaaiWabeaacqaIXaqmcqaI3aWnaiaawUhacaGL9baaaaa@8977@

It is evident from the linear stability analysis that un-damped oscillations exist there and that the steady state is a centre. This implies that system with stress-induced exercise is structurally stable.

**Figure 8:**
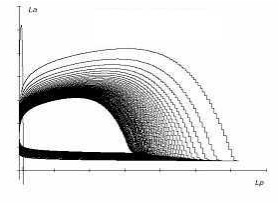
*L*_*a *_and *L*_*p *_interactions with concomitant stress and exercise; *h *= *0.1*.

**Figure 9:**
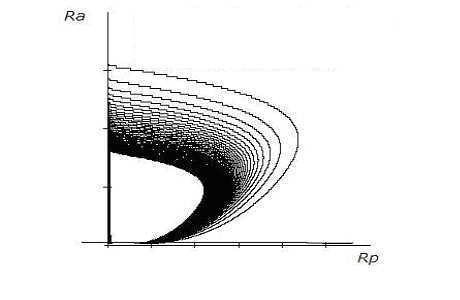
*R*_*a *_and *R*_*p *_interactions with concomitant stress and exercise and *h *= *0.1*.

Numerical solutions of system {15} are shown in figure 8 and figure 9; the values of the parameters are given Table 2. MATHCAD 13 computer software was used to obtain these numerical solutions.

To solve system {15} we used the fourth-order Runge-Kutta fixed-step method for solving systems of differential equations.

**Figure 7:**
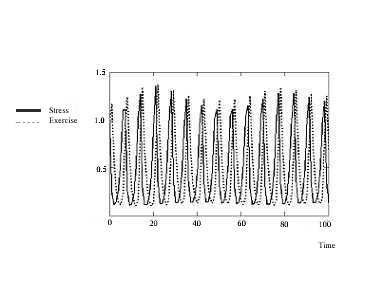
Oscillatory nature of stress (solid) and exercise (dotted).

The oscillatory behavior in response to concomitant stress and exercise (in any one quadrant, here LA) is depicted in Figure 7. The oscillatory nature of the behavior of two antero-posterior quadrants on the same side (left), *L*_*p *_and *L*_*a*_, is given in Figure 8. Similarly, the oscillatory nature of the behavior of two antero-posterior quadrants on the same side (right), *R*_*p *_and *R*_*a*_, is presented in Figure 9.

The outcomes of this model (where stress and exercise act together) show that there is a perfect harmony between the lateral hemispheric divisions of brain by exhibiting limit cycle solutions for (*L*_*p*_, *L*_*a*_) and (*R*_*p*_, *R*_*a*_). In this situation neither *L*_*p *_nor *L*_*a *_(and neither *R*_*a *_nor *R*_*p*_) can reach the maximum sustainable stage. Therefore, depression may not be manifested.

To extrapolate the behavior of this model biologically, it is highly possible that regular exercise in a moderate dose can counteract the lateralized effects of chronic stressors, over a substantial period of time, without allowing the harmful effects of stressors to take the upper hand.

### Model-3: The effects of different receptor subtype activities/concentrations in the different quadrants with stress and exercise

This is the most important part of this paper. We wanted to see whether our assumptions could lead to valid behavioral outcomes in real life.

Here we consider the activities/concentrations of the neurotransmitter receptor subtypes within *L*_*p*_, *L*_*a*_, *R*_*p *_and *R*_*a*_. As shown in Figures 3 and 6, Stress increases nt_s _activity/concentration in the *R*_*a *_and *L*_*p *_areas of the brain, and/or reduces nt_h _activity/concentration in the *L*_*a *_and *R*_*p *_areas, leading to a sad state. Exercise has the converse effects and elicits a happy mood.

We denote the activities/concentrations of these neurotransmitter receptor subtypes in the *L*_*p *_and *R*_*a *_regions by *C *and those in the *R*_*p *_and *L*_*a *_regions by *G*.

The time-dependent changes in activity/concentration may be modeled by the following equations.

dCdT=α11C(Ω−G)−β11CdGdT=α22G(C−Ω)−β22G     {18}
 MathType@MTEF@5@5@+=feaafiart1ev1aaatCvAUfKttLearuWrP9MDH5MBPbIqV92AaeXatLxBI9gBaebbnrfifHhDYfgasaacH8akY=wiFfYdH8Gipec8Eeeu0xXdbba9frFj0=OqFfea0dXdd9vqai=hGuQ8kuc9pgc9s8qqaq=dirpe0xb9q8qiLsFr0=vr0=vr0dc8meaabaqaciaacaGaaeqabaqabeGadaaakeaafaqaaeGabaaabaWaaSaaaeaacqWGKbazcqWGdbWqaeaacqWGKbazcqWGubavaaGaeyypa0JaeqySde2aaSbaaSqaaiabigdaXiabigdaXaqabaGccqWGdbWqcqGGOaakcqqHPoWvcqGHsislcqWGhbWrcqGGPaqkcqGHsislcqaHYoGydaWgaaWcbaGaeGymaeJaeGymaedabeaakiabdoeadbqaamaalaaabaGaemizaqMaem4raCeabaGaemizaqMaemivaqfaaiabg2da9iabeg7aHnaaBaaaleaacqaIYaGmcqaIYaGmaeqaaOGaem4raCKaeiikaGIaem4qamKaeyOeI0IaeuyQdCLaeiykaKIaeyOeI0IaeqOSdi2aaSbaaSqaaiabikdaYiabikdaYaqabaGccqWGhbWraaGaaCzcaiaaxMaadaGadeqaaiabigdaXiabiIda4aGaay5Eaiaaw2haaaaa@5D9A@

Where Ω is the neurotransmitter receptor subtype threshold level; α_11_, α_22 _and β_11_, β_22 _are the growth and decay parameters of neurotransmitter receptors sub-types.

If stress increases, eventually *nt*_*s *_activity/concentration in *L*_*p *_and *R*_*a *_regions increases at the same time *nt*_*h *_as activity/concentration in *L*_*a *_and *R*_*p *_regions decreases. Therefore, dCdT
 MathType@MTEF@5@5@+=feaafiart1ev1aaatCvAUfKttLearuWrP9MDH5MBPbIqV92AaeXatLxBI9gBaebbnrfifHhDYfgasaacH8akY=wiFfYdH8Gipec8Eeeu0xXdbba9frFj0=OqFfea0dXdd9vqai=hGuQ8kuc9pgc9s8qqaq=dirpe0xb9q8qiLsFr0=vr0=vr0dc8meaabaqaciaacaGaaeqabaqabeGadaaakeaadaWcaaqaaiabdsgaKjabdoeadbqaaiabdsgaKjabdsfaubaaaaa@319E@ ≥ 0 and dGdT
 MathType@MTEF@5@5@+=feaafiart1ev1aaatCvAUfKttLearuWrP9MDH5MBPbIqV92AaeXatLxBI9gBaebbnrfifHhDYfgasaacH8akY=wiFfYdH8Gipec8Eeeu0xXdbba9frFj0=OqFfea0dXdd9vqai=hGuQ8kuc9pgc9s8qqaq=dirpe0xb9q8qiLsFr0=vr0=vr0dc8meaabaqaciaacaGaaeqabaqabeGadaaakeaadaWcaaqaaiabdsgaKjabdEeahbqaaiabdsgaKjabdsfaubaaaaa@31A6@ ≤ 0.

Vice versa, if exercise increases, eventually *nt*_*h *_activity/concentration in *L*_*a *_and *R*_*p *_regions increases at the same time *nt*_*s *_as activity/concentration in *L*_*p *_and *R*_*a *_region decreases. Therefore, dCdT
 MathType@MTEF@5@5@+=feaafiart1ev1aaatCvAUfKttLearuWrP9MDH5MBPbIqV92AaeXatLxBI9gBaebbnrfifHhDYfgasaacH8akY=wiFfYdH8Gipec8Eeeu0xXdbba9frFj0=OqFfea0dXdd9vqai=hGuQ8kuc9pgc9s8qqaq=dirpe0xb9q8qiLsFr0=vr0=vr0dc8meaabaqaciaacaGaaeqabaqabeGadaaakeaadaWcaaqaaiabdsgaKjabdoeadbqaaiabdsgaKjabdsfaubaaaaa@319E@ ≤ 0 and dGdT
 MathType@MTEF@5@5@+=feaafiart1ev1aaatCvAUfKttLearuWrP9MDH5MBPbIqV92AaeXatLxBI9gBaebbnrfifHhDYfgasaacH8akY=wiFfYdH8Gipec8Eeeu0xXdbba9frFj0=OqFfea0dXdd9vqai=hGuQ8kuc9pgc9s8qqaq=dirpe0xb9q8qiLsFr0=vr0=vr0dc8meaabaqaciaacaGaaeqabaqabeGadaaakeaadaWcaaqaaiabdsgaKjabdEeahbqaaiabdsgaKjabdsfaubaaaaa@31A6@ ≥ 0. Here, the threshold level Ω plays the pivotal role.

**Figure 10:**
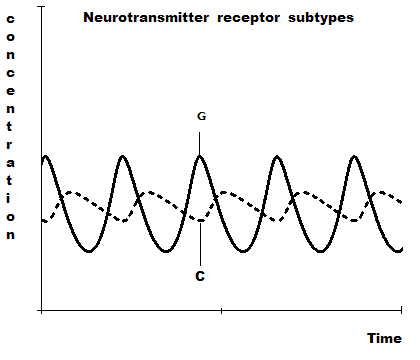
Oscillatory behavior of receptor subtype distributions in stress and exercise.

Numerical solutions of system {18} are shown in figure 10 and the parameter values are chosen arbitrarily (since experimental data are not available). MATHCAD 13 computer software was used to obtain these numerical solutions. Figure 10 compares their relative behaviors over time.

#### Parameter Choice

**Figure 11:**
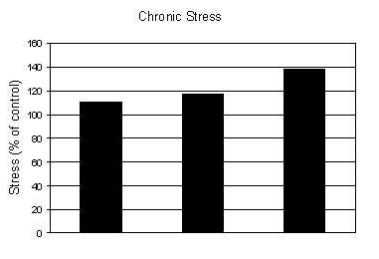
Development of chronic stress among rats based on High Plus Maze (HPM) experiment [1, 44].

**Figure 12:**
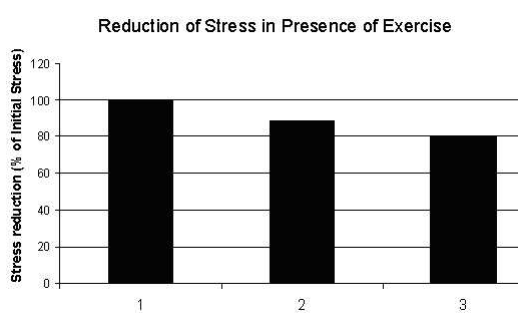
Reduction of stress due exercise among rats based on High Plus Maze (HPM) experiment [1, 44].

**Figure 13:**
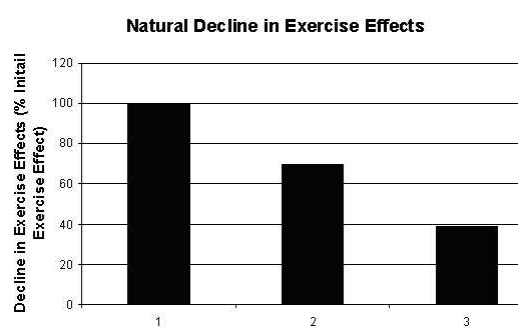
Natural Decline in Exercise effects among rats based on High Plus Maze (HPM) experiment [1, 44].

**Figure 14:**
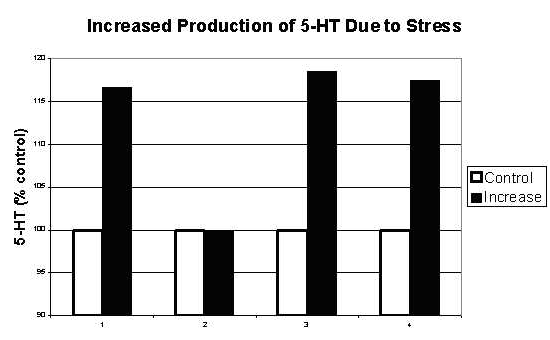
Increased in 5-HT due stress (15 min. forced swimming) among Wistar rats [48].

The experimental work of Sarbadhikari [1,44] on rats show that the response of exercise on stress, with respect to time, is reflected by a behavioral test such as High Plus Maze (HPM). On the basis of these behavioral studies, we have developed the following graphs (figures 11, 12, 13, 14) for chronic stress development, decline of stress due to exercise and normal degradation of the effects of exercise.

From figure 11, it is evident that in the case of chronic depression (stress), the growth curve of stress generation follows an exponential path:

*Stress *= 108.61 × *e*^0.016 × *time *^    {19}

Similarly, from figure 12, we find the decay in stress development due to the exercise as follows:

{DecayinStress}=99.783×e−0.0164×time     {20}
 MathType@MTEF@5@5@+=feaafiart1ev1aaatCvAUfKttLearuWrP9MDH5MBPbIqV92AaeXatLxBI9gBaebbnrfifHhDYfgasaacH8akY=wiFfYdH8Gipec8Eeeu0xXdbba9frFj0=OqFfea0dXdd9vqai=hGuQ8kuc9pgc9s8qqaq=dirpe0xb9q8qiLsFr0=vr0=vr0dc8meaabaqaciaacaGaaeqabaqabeGadaaakeaadaGadeqaauaabeqadeaaaeaacqWGebarcqWGLbqzcqWGJbWycqWGHbqycqWG5bqEaeaacqWGPbqAcqWGUbGBaeaacqWGtbWucqWG0baDcqWGYbGCcqWGLbqzcqWGZbWCcqWGZbWCaaaacaGL7bGaayzFaaGaeyypa0JaeGyoaKJaeGyoaKJaeiOla4IaeG4naCJaeGioaGJaeG4mamJaey41aqRaemyzau2aaWbaaSqabeaacqGHsislcqaIWaamcqGGUaGlcqaIWaamcqaIXaqmcqaI2aGncqaI0aancqGHxdaTcqWG0baDcqWGPbqAcqWGTbqBcqWGLbqzaaGccaWLjaGaaCzcamaacmqabaGaeGOmaiJaeGimaadacaGL7bGaayzFaaaaaa@5E59@

From figure 13, the natural degradation of exercise is as follows:

{Naturaldecayofexercise}=103.78×e−0.0681×time     {21}
 MathType@MTEF@5@5@+=feaafiart1ev1aaatCvAUfKttLearuWrP9MDH5MBPbIqV92AaeXatLxBI9gBaebbnrfifHhDYfgasaacH8akY=wiFfYdH8Gipec8Eeeu0xXdbba9frFj0=OqFfea0dXdd9vqai=hGuQ8kuc9pgc9s8qqaq=dirpe0xb9q8qiLsFr0=vr0=vr0dc8meaabaqaciaacaGaaeqabaqabeGadaaakeaadaGadeqaauaabeqaeeaaaaqaaiabd6eaojabdggaHjabdsha0jabdwha1jabdkhaYjabdggaHjabdYgaSbqaaiabdsgaKjabdwgaLjabdogaJjabdggaHjabdMha5bqaaiabd+gaVjabdAgaMbqaaiabdwgaLjabdIha4jabdwgaLjabdkhaYjabdogaJjabdMgaPjabdohaZjabdwgaLbaaaiaawUhacaGL9baacqGH9aqpcqaIXaqmcqaIWaamcqaIZaWmcqGGUaGlcqaI3aWncqaI4aaocqGHxdaTcqWGLbqzdaahaaWcbeqaaiabgkHiTiabicdaWiabc6caUiabicdaWiabiAda2iabiIda4iabigdaXiabgEna0kabdsha0jabdMgaPjabd2gaTjabdwgaLbaakiaaxMaacaWLjaWaaiWabeaacqaIYaGmcqaIXaqmaiaawUhacaGL9baaaaa@6AA6@

The above three equations {19}–{21} give us the approximate values of four parameters ξ_5 _= 0.016, ς_5 _= 0.0164, ξ_6 _= 0.0164 and ς_6 _= 0.0681 with 0.94 ≤ *R*^2 ^≤ 0.99.

The rest of the parameters of the system are calculated on the basis of the experimentally reported data mentioned below.

The steady states of dimensionless stress and exercise are given by

{x5s
 MathType@MTEF@5@5@+=feaafiart1ev1aaatCvAUfKttLearuWrP9MDH5MBPbIqV92AaeXatLxBI9gBaebbnrfifHhDYfgasaacH8akY=wiFfYdH8Gipec8Eeeu0xXdbba9frFj0=OqFfea0dXdd9vqai=hGuQ8kuc9pgc9s8qqaq=dirpe0xb9q8qiLsFr0=vr0=vr0dc8meaabaqaciaacaGaaeqabaqabeGadaaakeaacqWG4baEdaqhaaWcbaGaeGynaudabaGaem4Camhaaaaa@30B9@ = 4.15,     x6s
 MathType@MTEF@5@5@+=feaafiart1ev1aaatCvAUfKttLearuWrP9MDH5MBPbIqV92AaeXatLxBI9gBaebbnrfifHhDYfgasaacH8akY=wiFfYdH8Gipec8Eeeu0xXdbba9frFj0=OqFfea0dXdd9vqai=hGuQ8kuc9pgc9s8qqaq=dirpe0xb9q8qiLsFr0=vr0=vr0dc8meaabaqaciaacaGaaeqabaqabeGadaaakeaacqWG4baEdaqhaaWcbaGaeGOnaydabaGaem4Camhaaaaa@30BB@ = 0.98}.

#### Parameter estimations for *L*_*P*_, *L*_*a *_and *R*_*a*_, *R*_*P *_dynamics

From De La Garza and Mahoney's [48] experimental work it is evident that there is a stress-induced increase of 5-HT (Serotonin – a neurotransmitter) concentration among Wistar rats in all sectors of brains (*mPFCtx*, *NAS*, *Amygdala *except *Striatum*). Figure 14 shows the increase in 5-HT concentrations in different sectors of brain.

Here we assume that the secretion of 5-HT reaches the steady state level owing to 15 minutes' forced swimming activity and the intrinsic growth rate of 5-HT (synthesis rate – decay rate) is equivalent to the characteristic time (τs=115)
 MathType@MTEF@5@5@+=feaafiart1ev1aaatCvAUfKttLearuWrP9MDH5MBPbIqV92AaeXatLxBI9gBaebbnrfifHhDYfgasaacH8akY=wiFfYdH8Gipec8Eeeu0xXdbba9frFj0=OqFfea0dXdd9vqai=hGuQ8kuc9pgc9s8qqaq=dirpe0xb9q8qiLsFr0=vr0=vr0dc8meaabaqaciaacaGaaeqabaqabeGadaaakeaadaqadiqaaiabes8a0naaBaaaleaacqWGZbWCaeqaaOGaeyypa0ZaaSaaaeaacqaIXaqmaeaacqaIXaqmcqaI1aqnaaaacaGLOaGaayzkaaaaaa@358F@.

Based on this assumption we obtain the following parameters values:

ξ_1 _= 0.068, ς_1 _= 0.0012, ξ_4 _= 0.68 and ς_4 _= 0.0012

In a similar way we approximate the parameter values related to the effects of exercise in *L*_*a *_and *R*_*p *_sections of the brain from the experimental data reported by Gomez-Pinilla *et al*. [49] on exercise-induced BDNF-mediated mechanisms. These authors suggest that there is a significant increase of BDNF mRNA due to voluntary running on an exercise apparatus with loads for seven days. On the basis of these data we have estimated the following parameters:

ξ_2 _= 0.143, ς_2 _= 0.00014, ξ_3 _= 0.143 and ς_3 _= 0.00014

Table 2 gives all the parameter ranges

The threshold parameter *h *varies over the range 0.1 to 1.00, depending upon the individual's neuron threshold capacity. The cooperative constants *n*_*i*_(*i *= 1,2,3,4) are assumed to be unity. For Model-3, the parameter values are: Ω = (0.001/0.016), α_11 _= 0.016, α_22 _= 0.016, β_11 _= 0.016 and β_22 _= 0.068.

### Summary of the hypothetical models

The models described in this article show one of the likely mechanisms for the action, on the brain, of concomitant stress and exercise. They mathematically prove our assumptions that 'Stress' nonlinearly increases the activity/concentration of particular subtypes of receptors (designated *nt*_*s*_) for each of the known (and unknown) neurotransmitters in the right anterior (RA or *R*_*a*_) and left posterior (LP or *L*_*p*_) regions of the brain, and/or nonlinearly decreases the activity/concentration of another set of receptor subtypes (designated *nt*_*h*_) for each of these neurotransmitters in the left anterior (LA or *L*_*a*_) and right posterior (RP or *R*_*p*_) activity areas. Exercise elicits the opposite (nonlinear) effects.

In other words, the behavior of the models can be experimentally verified by suitable designs, as outlined in the section on implications of the hypothesis.

## Limitations

Zaldivar *et al*. [50] have shown that exercise increases circulation of the same pro-inflammatory cytokines that are normally upregulated during a response to stress. However, exercise may also upregulate anti-inflammatory cytokines, and over time increases the threshold for the immune system responses to stress. The present model does not take into account the bimodal (hormetic) action of exercise, based on the other studies cited earlier [11,23,36,39,44]. This has already been mentioned in the background section.

It is established that the intensity of exercise, the level of fitness, and various individual differences (which may be due to "nature" or "nurture"), impact acute affective responses to exercise [51,52]. Also, Bixby *et al*. [51] have shown that exercise intensity impacts the affective response during and after exercise, with higher intensity exercise being associated with more negative affect during exercise. These aspects will be dealt with in the future models.

Furthermore, exercise can also influence other brain parameters such as blood flow, antioxidant activities, neuronal apoptosis, receptor sensitization, glutamate secretion and many other unknown factors, which in various combinations can have some effect on depression. Our model does not explicitly deal with any of these effects in detail.

Dishman *et al*. [53] write: "Chronic voluntary physical activity also attenuates neural responses to stress in brain circuits responsible for regulating peripheral sympathetic activity, suggesting constraint on sympathetic responses to stress that could plausibly contribute to reductions in clinical disorders such as hypertension, heart failure, oxidative stress, and suppression of immunity. Mechanisms explaining these adaptations are not as yet known, but metabolic and neurochemical pathways among skeletal muscle, the spinal cord, and the brain offer plausible, testable mechanisms that might help explain effects of physical activity and exercise on the central nervous system." Our model provides one possible direction towards solving some of the puzzles.

Greenwood *et al*. [54] suggest that the central 5-HT system is sensitive to wheel running in a time-dependent manner. The observed changes in mRNA regulation in a subset of raphe nuclei might contribute to the stress resistance produced by wheel running and the antidepressant and anxiolytic effects of physical activity. We believe that more than one or two neurotransmitter systems are simultaneously involved in leading to the observed nonlinear behaviors of stress and exercise.

## Implications of the hypothesis

To sum up the information gathered in this paper, we can see that many antidepressive interventions exert their therapeutic effects through various neurotransmitters, mainly acting nonlinearly through their several specific receptor subtypes (Figure 3). The final common pathway (biological cascade) is at the cellular and subcellular levels. Therefore, to achieve therapeutic benefit, lower level targets are now being selected, *e.g*., adenosine (A_2A_) and calcium (Ca^2+^) channels, as well as genes for BDNF and CREB. The underlying neural networks function on the basis of the inputs received from the various neurotransmitter receptor subtypes. Detailed expositions are given elsewhere [26,27].

Experiments may be devised to measure changes in concentrations and activity levels of various neurotransmitters and of growth factors such as BDNF in different regions of the brain, followed by identification of specific receptor subtypes in these regions. It may be noted that it is beyond the capacity of a single researcher or even a single group to validate all the experimental possibilities predicted from our model. We enumerate a few of these possibilities below.

1. Choose any neurotransmitter and verify the differences in activity and concentration of its receptor subtypes in different parts of the brain – during healthy condition, with regular moderate physical exercise, with chronic stress, and various combinations of these conditions.

2. Similar experiments may be devised for all other neurotransmitters.

3. Measure the changes in concentration/activity of BDNF and/or other neurotrophic factors during the above conditions and their possible correlation with neurotransmitter receptor subtype activity in the specific regions of the brain.

4. Correlate the neurochemical findings with functional (fMRI) and quantitative EEG findings.

5. Correlate clinical conditions with the laboratory findings and identify specific target areas for effective drug design.

6. Translate the experimental findings into effective pharmacological/non-pharmacological (e.g., regular moderate exercise) interventions.

7. Try further mathematical modeling and refinement based on newer experimental evidence.

In addition, automated techniques [55,56] may be applied for modeling and simulating the experimental findings *in-silico *and leading to specific predictions and further refinements of the experimental procedures.

On the basis of this hypothesis, a general model incorporating the oscillation caused by the same neurotransmitter acting on different receptor subtypes, and with the pattern of recruitment of these subtypes over time, may lead to a better understanding of brain neurodynamics. Well-designed practical experiments will serve to test such theoretical models and shed more light on the underlying brain dynamics.

## Other future trends (postscript)

The extensor motor system may be particularly important in the theory of mind (psychomotor theory) including some neural disorders such as depression [57]. Accordingly, an increase in extensor motor system activity is likely to be beneficial for the treatment of depression. Our hypothesis may incorporate this new theory in future.

Other factors are involved in mediating the effects of exercise in depression. Sleep deprivation has a negative effect on cognitive and psychomotor performance and mood state, partially because of decreased creatine levels in the brain. Recently, a study [58] designed to examine the effect of creatine supplementation and sleep deprivation, with mild exercise, on cognitive and psychomotor performance, mood state, and plasma concentrations of hormones showed that norepinephrine and dopamine concentrations were significantly higher after 24 h sleep deprivation than at 0 h, but cortisol levels were lower. This study suggested that after 24 h sleep deprivation, creatine supplementation had a positive effect on mood state and tasks that place a heavy stress on the prefrontal cortex.

In another recent study [59], concomitant diet regulation and exercise were shown to reduce muscle sympathetic nerve activity during mental stress.

Two other recent papers [60,61] endorse the role of distinct receptor subtypes in mediating the anti-depressant effects of exercise. The CNS effects of running are different in 'depressed' and control animals [61]. NPY (Neuropeptide Y) is associated with depression and anxiety neurotransmission in hippocampal malfunctions in depression, and antidepressive treatment (wheel running) normalizes its level. In addition, these authors [61] show that the increase in NPY after running is correlated with increased cell proliferation, which is associated with an antidepressive-like effect. The role of differential modulation by distinct subtypes of a neurotransmitter receptor depends on the initial condition and the patent connectivity between the diverse networks within the brain [62].

Repeated measures of multivariate analysis of variance (MANOVA) have been used to test for differences in N200 and P300 amplitude of evoked potentials between SD (subclinical depression) and ND (no depression or normal) groups [63]. ND, but not SD, groups show asymmetry (R>L) in central N200 amplitude. Similar asymmetry is seen in ND, but not SD, men at posterior sites. SD groups demonstrate left>right posterior P300 amplitude asymmetry owing to P300 enhancement at left temporoparietal sites. Results support involvement of various cognitive mechanisms measured by P300 and N200 in subclinical depressive symptoms, some of which may rely on sex. We find that various investigators around the globe were actually validating different parts of our speculative model while we were preparing this paper.

The purpose of citing schizophrenia in various parts of this paper is to show the similarities between apparently unrelated disorders making use of the same brain circuitry, albeit in variable strengths. As already mentioned, some of the stress responses may be similar in such different conditions as major depression, depressive phase of bipolar disorder and schizophrenia. Recent experiments also confirm such concepts, as in [64]. These authors showed that various convergent cytogenetic and genetic findings provide molecular evidence for common etiologies for different psychiatric conditions such as bipolar disorder and schizophrenia and further support the 'glutamate hypothesis' of psychotic illness. Earlier, too, [65] there were subtle indications. We sincerely hope that our model will be able to integrate various such disciplines in the search for a comprehensive mechanism of action for chronic moderate exercise and chronic stress acting through the different regions of the brain.

## Conclusion

Etevenon [66] proposed, more than two decades ago, a model for cross-coupling of diagonal quadrants of the brain in affect processing – but there was hardly any empirical data to support the proposal. With the advance of technology and its applications in the healthcare domain we are in a better position to construct a more realistic model.

Numerous other contemporary scientists [67,68] have demonstrated that mathematical modeling is a useful tool for diagnosing and assessing the prognosis of depression.

Future models are bound to be modified and refined as more and more experimental evidence is gathered owning to advances in technology. We have tried to integrate diverse domains of knowledge about depressive disorders and exercise physiology. It may not currently be possible to test the hypothesis holistically, but there is an immediate need for domain experts to come together from various disciplines such as neuropsychology, computational neuroscience, exercise science, molecular biology, clinical psychophysiology, bedside clinics, experimental neurophysiology, behavior therapy and nonlinear dynamics. The necessity for this theoretical modeling arose because of the lack of experimental data relating to all aspects of our hypothesis. We hope that by using the outcomes of these models, experimental biologists will be able to devise experiments involving diverse subtypes of the same neurotransmitters, acting differently in localized areas of the brain (in health and disease), reinforce (or refute) our assumptions, and enable more refined and practically applicable versions of the present hypothesis to be elaborated.

## Competing interests

The author(s) declare that they have no competing interests.

## Authors' contributions

The entire theoretical concept of the work has been envisaged by SNS. The mathematical modeling has been carried out by AKS with feedback from SNS.
